# The ubiquitin-specific protease 21 is critical for cancer cell mitochondrial function and regulates proliferation and migration

**DOI:** 10.1016/j.jbc.2024.107793

**Published:** 2024-09-19

**Authors:** Magdalena Kulma, Bartłomiej Hofman, Małgorzata Szostakowska-Rodzoś, Dorota Dymkowska, Remigiusz A. Serwa, Katarzyna Piwowar, Agnieszka Belczyk-Ciesielska, Joanna Grochowska, Irina Tuszyńska, Angelika Muchowicz, Katarzyna Drzewicka, Krzysztof Zabłocki, Zbigniew Zasłona

**Affiliations:** 1 Molecure SA, Warsaw, Poland; 2 Laboratory of Cellular Metabolism, Nencki Institute of Experimental Biology, Polish Academy of Sciences, Warsaw, Poland; 3 IMol, Polish Academy of Sciences, Warsaw, Poland; 4 ReMedy International Research Agenda Unit, IMol, Polish Academy of Sciences, Warsaw, Poland

**Keywords:** ATP, bioenergy, cancer metabolism, deubiquitination, STAT3, USP21

## Abstract

Ubiquitin-specific proteases (USPs) are the main members of deubiquitinases (DUBs) that catalyze removing ubiquitin chains from target proteins, thereby modulating their half-life and function. Enzymatic activity of USP21 regulates protein degradation which is critical for maintaining cell homeostasis. USP21 determines the stability of oncogenic proteins and therefore is implicated in carcinogenesis. In this study, we investigated the effect of USP21 deletion on cancer cell metabolism. Transcriptomic and proteomic analysis of USP21 KO HAP-1 cells revealed that endogenous USP21 is critical for the expression of genes and proteins involved in mitochondrial function. Additionally, we have found that the deletion of USP21 reduced STAT3 activation and STAT3-dependent gene and protein expression in cancer cells. Genetic deletion of USP21 impaired mitochondrial respiration and disturbed ATP production. This resulted in cellular consequences such as inhibition of cell proliferation and migration. Presented results provide new insights into the biology of USP21, suggesting novel mechanisms for controlling STAT3 activity and mitochondrial function in tumor cells. Taken together, our findings indicate that targeting USP21 dysregulates the energy status of cancer cells offering new perspectives for anticancer therapy.

Protein homeostasis is critical for maintaining proper cellular function and is tightly regulated by several processes, including protein synthesis, folding, trafficking, and stability of proteins. Both ubiquitination and deubiquitination regulate the stability of proteins essential for cellular function (1, 2, 3). Ubiquitin-specific proteases (USPs) belong to a large group of deubiquitylating enzymes (deubiquitinases, DUBs) that catalyze the cleavage of ubiquitin chains from ubiquitinylated target proteins, protecting them from proteasomal degradation (4). Deubiquitylation is a posttranslational modification critical for diverse cellular processes, such as immune signaling, DNA repair pathways, cell apoptosis, as well as cell cycle, and division (5). Overexpression of USPs can cause dramatic physiological consequences that lead to cancer development and progression (5, 6, 7, 8, 9), as well as neurodegenerative and inflammatory diseases (10, 11) and therefore, USP family members are considered as novel therapeutic targets.

Ubiquitin specific protease 21 (USP21) is a member of USPs with elevated expression in several cancers, including pancreatic, non–small cell lung, renal, and breast cancers (9, 12, 13, 14). Experimental evidence showed that upregulation of USP21 promotes cancer progression by stabilization of transcription factors crucial for tumor growth and metastasis (9, 12, 15, 16, 17). One of the most important oncogenic transcription factors engaged in a regulation of the signaling pathway associated with cancer is signal transducer and activator of transcription 3 (STAT3) (18). STAT3 is a cytoplasmic transcription factor that undergoes phosphorylation leading its dimerization, translocation into the nucleus, and binding to the regulatory sequence of targeted genes (19). Constitutive activation and overexpression of STAT3 have been reported in nearly 70% of cancers (20) and are responsible for maintaining cell growth, migration, proliferation, metastasis, and survival (21, 22, 23). As a consequence of regulating multiple signaling pathways crucial for cancer progression, STAT3 has been considered as an attractive therapeutic target. Recent studies have extended our understanding of STAT3 beyond the regulation of oncogenesis, showing its pleiotropic effects on a spectrum of fundamental cellular processes, such as mitochondrial function, including energy transduction, mitochondrial gene expression, as well as oncogenic cellular metabolism (24, 25, 26). Mitochondria provide key metabolites for tumor anabolism, control redox, and calcium homeostasis playing an essential role in cancer cell migration and tumor development (27, 28). Therefore, novel approaches to target mitochondria for the development of anticancer agents are emerging (29, 30). Currently, two types of STAT3 inhibitors have been developed: direct binders to STAT3 domains and indirect, which inhibit the STAT3 signaling upstream by targeting JAKs, Src, and Abl. Despite many efforts, effective therapeutic interventions directly targeting STAT3 to achieve potent antitumor effects in the clinic have not been identified and still need to be elucidated (31). While, the inhibitors of JAKs are successfully used in the treatment of cancer and immune-mediated diseases (32), suggesting that indirect inhibition of STAT3 might be a more promising approach.

Here, we demonstrate for the first time that downregulation of USP21inhibits mitochondrial respiration and oxidative phosphorylation in cancer cells. Disruption of cellular metabolism triggered by USP21 deletion proves that pharmaceutical inhibition of USP21 may decrease mitochondrial metabolism, leading to inhibition of cancer progression. Thus, our results strongly suggest that the USP21-dependent STAT3-mediated metabolic pathway is a promising target for anticancer therapy.

## Results

### USP21 controls the expression of genes and proteins involved in mitochondrial function

**Figure 1:**
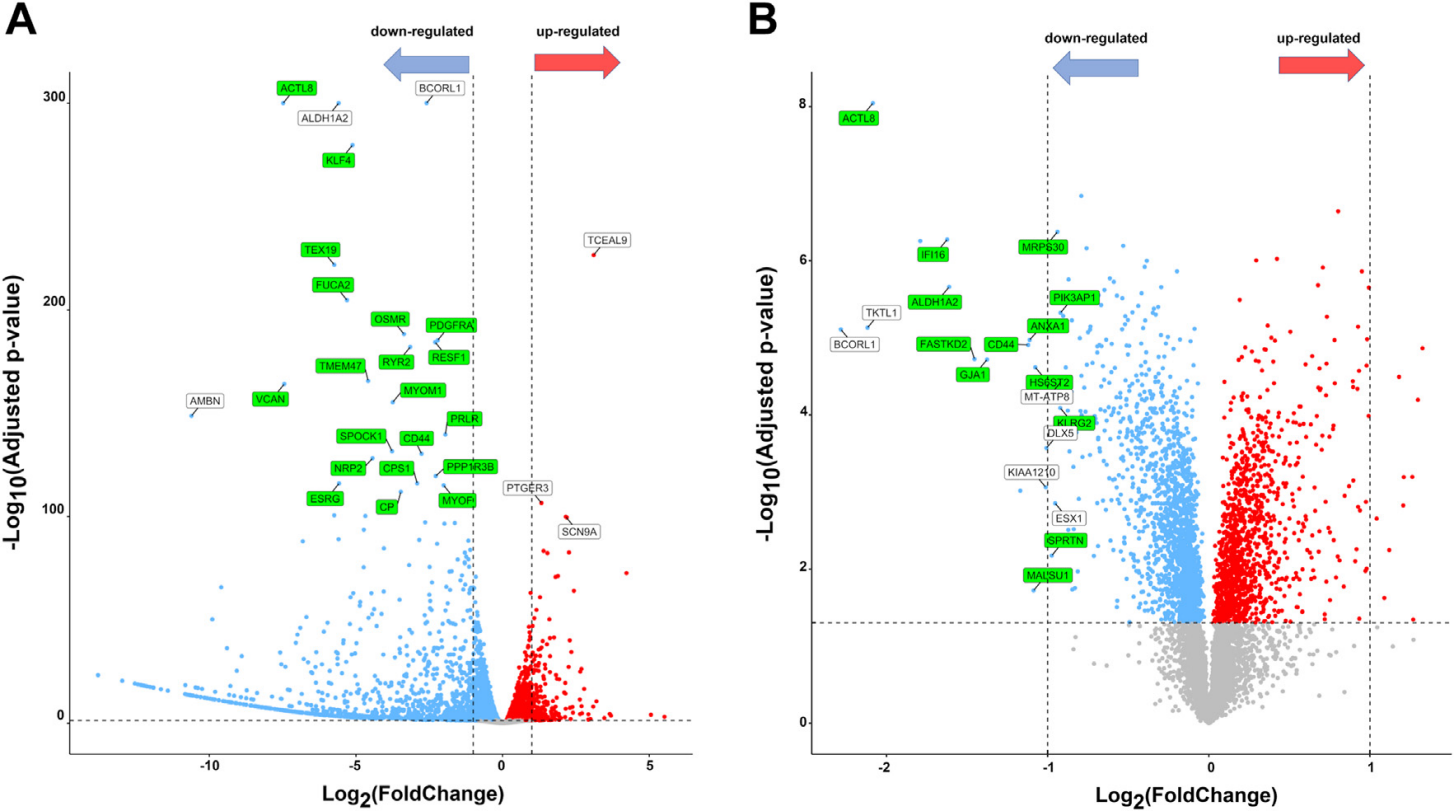
**Analysis of STAT3-dependent transcriptome and proteome changes in HAP-1 USP21 KO *versus* HAP-1 WT cells.** Comparative transcriptomic and proteomic analysis of USP21 KO HAP-1 cells *versus* control cells. Volcano plots show differentially expressed genes (*A*) and proteins (*B*) in USP21 KO HAP-1 cells *versus* parental HAP-1 cells. The data points above the significance threshold (q < 0.05) are marked in *red* (upregulated in USP21 KO) and *blue* (downregulated in USP21 KO). The *gray dashed vertical lines* indicate the thresholds of |log2(FoldChange)| = 1 and |log2(FoldChange)| = 0.5 for transcriptomic and proteomic data, respectively. Transcripts and proteins with insignificant changes are depicted in *gray*. Transcription factors regulating expression of 20 most significantly downregulated transcripts and the most downregulated proteins were analyzed using ChIP Enrichment Analysis (ChEA) and Encode databases and the names are marked on volcano plots. Transcripts and proteins regulated by STAT3 are marked on volcano plots in *green squares*, regulated in STAT3-independent way are marked in *gray squares*. STAT3, signal transducer and activator of transcription 3; USP, ubiquitin-specific protease

DUBs act as major regulators of a wide spectrum of cellular processes by targeting proteins for expression and proteasomal degradation (33). To determine the role of USP21 on cellular processes, an omics analysis was performed. The effect of USP21 deletion in cancer cells was studied in CRISPR/Cas9-edited USP21-targeting HAP-1 cells (HAP-1 USP21 KO) in comparison to HAP-1 parental control (HAP-1 WT). HAP-1 is a human chronic myelogenous leukemia-derived cell line (34), which due to its haploid genome is widely used in cancer research, genetic, and multiomics studies. Transcriptome analysis of differential gene expression revealed that deletion of USP21 leads to upregulation of 201 genes and downregulation of 1006 genes in HAP-1 cells (log2(fold change) > 1 and q-value < 0.05) (Fig. 1*A*; Table S1). This result suggests that USP21 regulates a plethora of cellular processes at the mRNA expression level.

Then, we searched the ENCODE and ChEA transcription factor databases to determine whether the down-expression of genes in HAP-1 USP21 KO cells resulted from regulation by a specific transcription factor. Bioinformatic analysis of binding site profiles datasets showed that a great majority of down-expressed genes in USP21 KO cells are regulated by STAT3 (Fig. 1*A*).

In the next step, we used mass spectrometry based proteomic analysis to reveal alterations in the level of proteins, resulting from USP21-dependent proteasomal degradation as well as USP21-dependent regulation of transcription factors (Fig. 1*B*). High-throughput mass spectrometry analysis identified 6455 proteins, showing, in contrast to transcriptomic data, a similar number of downregulated and upregulated proteins in HAP-1 USP21 KO cells. A *t* test statistical analysis showed that genetic deletion of USP21 significantly affected a HAP-1 cell proteome, upregulating 128 proteins (log2(fold change) > 0.5 and q-value < 0.05) and downregulating 149 proteins (log2(fold change) < −0.5 and q-value < 0.05) (Table S2). We found that decreased level of certain proteins such as ACTL8, FASTKD2, KLF4, ALDH1A2, and BCORL1 (Fig. 1*B*) corresponded to downregulated transcripts (Fig. 1*B*), suggesting that decreased level of these proteins could be a consequence of regulation by USP21-dependent transcription factors. For this reason, the top 20 down-expressed proteins in HAP-1 USP21 KO cells were verified for transcription factors controlling their expression. Similarly to transcriptomic analysis, it was found that 13 of them were regulated by STAT3 transcription factor (Fig. 1, *A* and *B*).

**Figure 2:**
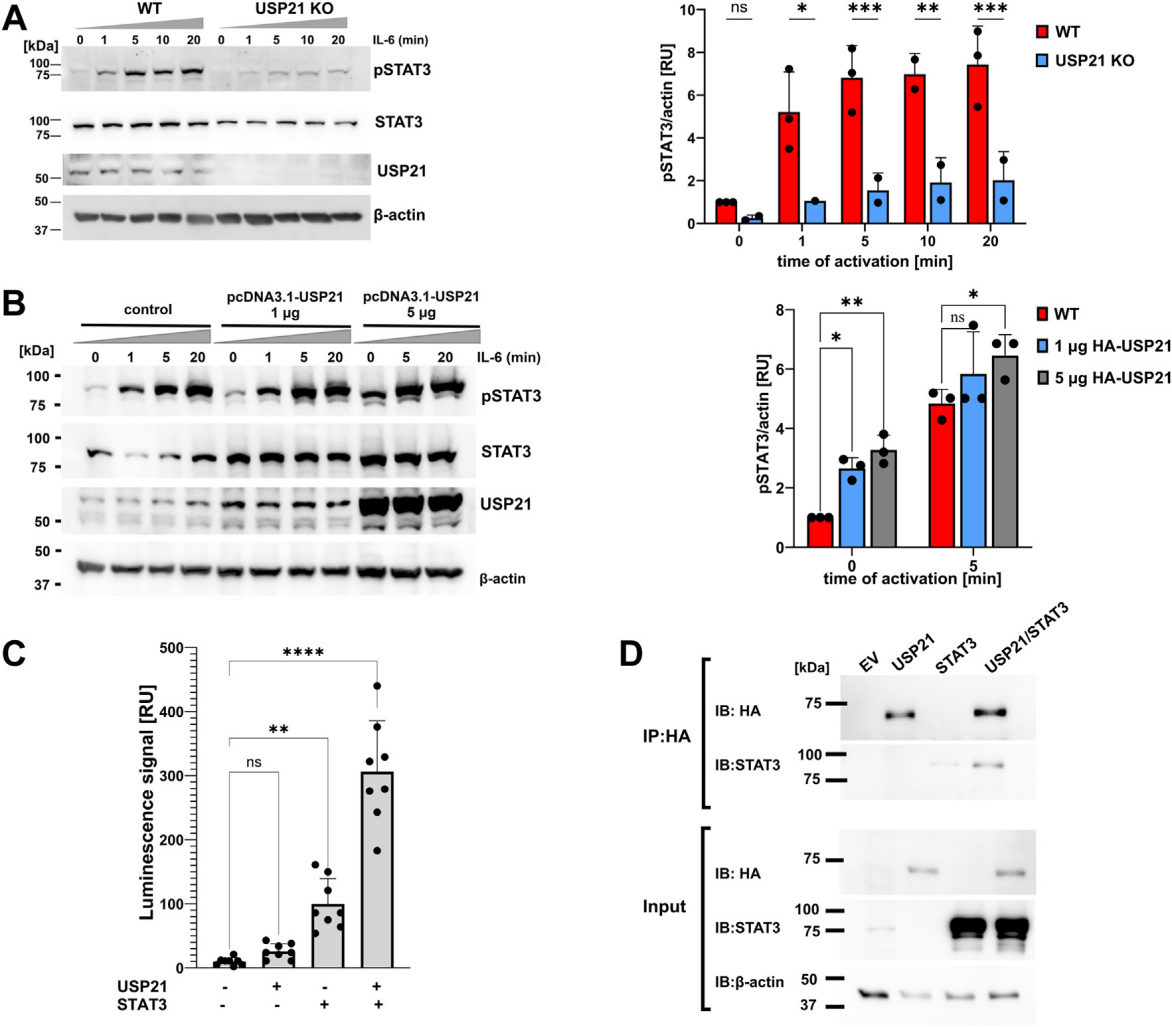
**USP21 is a positive regulator of STAT3 expression and phosphorylation in HAP-1 cells**. The effect of USP21 KO (*A*) and overexpression (*B*) on STAT3 expression level and Tyr705 phosphorylation in HAP-1 cells was analyzed by Western blot and detection of pSTAT3, STAT3, USP21, and β-actin. *A*, HAP-1 cells (parental and USP21 KO, 1 × 10^6^/sample) were treated with 50 ng/ml IL-6 for up to 20 min. Then cell lysates were used for Western blot analysis. The histogram (*right panel*) depicts the levels of pSTAT3 in HAP-1 WT *(red*) and HAP-1 USP21 KO (*blue*) determined through densitometry corrected for actin (*B*). For analysis of STAT3 phosphorylation in HAP-1 cells overexpressing USP21, cells were transfected with pcDNA3.1-USP21 vector (1 μg and 5 μg) by nucleofection. After 24 h cells were stimulated with IL-6 for 5 and 20 min. The level of pSTAT3, STAT-3, USP21, and β-actin was detected by Western blot. Histogram (*right panel*) represent relative mean band intensity of pSTAT3 for HAP-1 cells untransfected (*red*) and transfected with 1 μg (*blue*) and 5 μg (*gray*) of pcDNA3.1-USP21. The effect of USP21 overexpression on STAT3 activation analyzed by Dual-Glo luciferase system (*C*). pTF-STAT3-Luc, pcDNA3.1-HA-USP21, or pcDNA3.1 (EV; empty vector) plasmids were transfected in HEK293 cells for 8 h, followed by activation with IL-6 for 16 h. Then, the cells were subjected to luminescence measurement by using a Dual-Glo luminescence assay. Interaction of USP21 with STAT3 (*D*). HA-USP21 or STAT3 encoding plasmids were transfected into HEK293T for 48 h, and then cell lysates were prepared for coimmunoprecipitation and immunoblotting against specific antibodies as indicated. The standard deviation was determined from three independent repetitions. ∗*p* < 0.05, ∗∗*p* < 0.01, ∗∗∗*p* < 0.001, ∗∗∗∗*p* < 0.0001, ns indicates no statistical difference (*p* > 0.05). IL-6, interleukin 6; pSTAT3, Tyr705 phosphorylated signal transducer and activator of transcription 3; STAT3, signal transducer and activator of transcription 3; USP, ubiquitin-specific protease.

To validate the impact of USP21 on STAT3 activation, the level of STAT3 expression and STAT3 phosphorylation at tyrosine 705 (pSTAT3) was determined in interleukin 6 (IL-6)-stimulated HAP-1 WT and HAP-1 USP21 KO cells. We found that KO of USP21 inhibited STAT3 phosphorylation and affected total STAT3 level (Fig. 2*A*). These data suggest that USP21 is required for the expression of genes regulated by STAT3. In order to comprehensively address the regulation of STAT3 activity by USP21, we checked the consequence of USP21 overexpression in HAP-1 cells on a STAT3 expression and phosphorylation. We observed that an increased level of USP21 in HAP-1 cells enhanced phosphorylation of STAT3, both in IL-6- stimulated and unstimulated cells, as well as increased expression of STAT3 (Fig. 2*B*). Other IL-6-stimulated signaling pathways, such a phospho-protein kinase B and phospho-extracellular signal-regulated kinase were unchanged in HAP-1 WT and USP21 KO cells, indicating that our findings are specific for STAT3-mediated signaling pathway (Fig. S1). Additionally, the STAT3 luciferase reporter assay confirmed that USP21 regulates STAT3 signaling. As shown in Fig. 2*C*, overexpression of USP21 in HEK293T cells significantly (three times) increased STAT3-dependent luciferase activity. To determine if STAT3 interacts with USP21 directly, the coimmunoprecipitation assay was performed. Indeed, the overexpressed USP21 interacted with exogenous STAT3, suggesting regulation of STAT3 level in cells by its deubiquitination (Fig. 2*D*).

**Figure 3:**
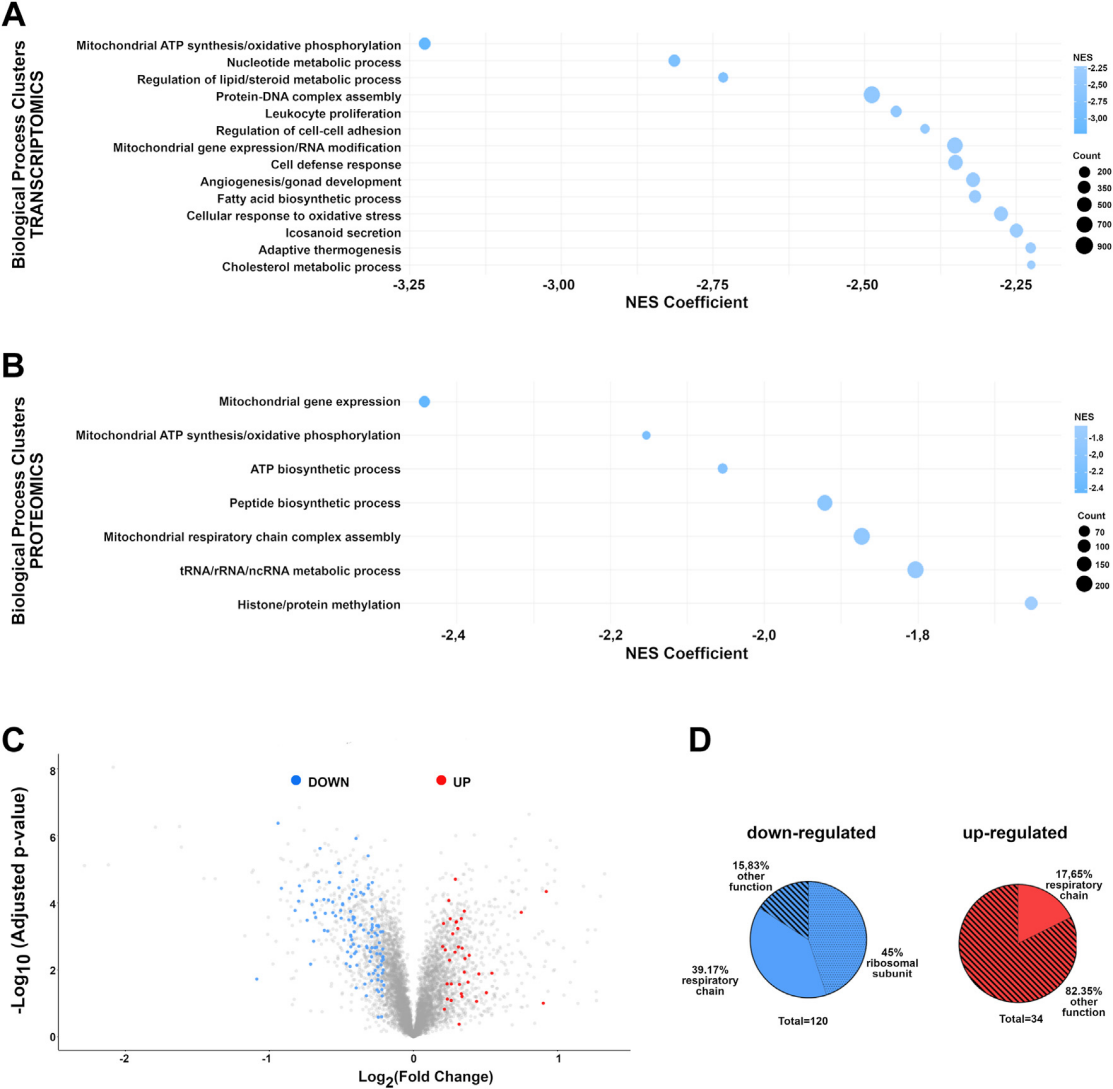
**USP21 is essential for mitochondrial functioning in HAP-1 cells.** Gene Set Enrichment Analysis (GSEA) for Gene Ontology (GO) biological processes from transcriptome and proteome analysis of the WT and USP21 KO cells (*A* and *B*). Selected biological processes of transcriptome (*A*) and proteome (*B*) analysis are presented as the clusters ranking list with respect to the median NES coefficient of a given cluster Negative NES indicates downregulation of the most leading genes from the cluster. The complete results of the GSEA analysis are presented in the Table S3, *A* and *B*. Dysregulated proteins involved in the functioning of mitochondria (according to MitoCarta3.0 classification) overlaid on volcano plot depicting relative differences of protein levels in total cell extracts from USP21 KO *versus* WT cells (*C*). Downregulated proteins are marked in *blue*, upregulated proteins are marked in *red*. Schematic presentation of downregulated and upregulated proteins involved in mitochondrial function in HAP-1 USP21 KO cells (*D*). Downregulated (*blue*) and upregulated (*red*) proteins in USP21 KO *versus* WT cells involved in mitochondrial function were selected from proteome analysis and clustered into three groups, depending on their function: ribosomal subunit assembly (*crossed*), respiratory chain (*plain*), and participated in another function of mitochondria (*striped*). ∗*p* < 0.05, ∗∗*p* < 0.01, ∗∗∗∗*p* < 0.0001, ns represents no statistical difference (*p* > 0.05). NES, normalized enrichment score; USP, ubiquitin-specific protease.

Subsequent analysis of transcriptomic and proteomic data characterized cellular processes triggered by a lack of endogenous USP21. Based on the Gene Ontology (GO) gene sets, we have found that several of the most downregulated proteins were involved in mitochondrial processes, including oxidative phosphorylation, aerobic respiration, mitochondrial gene expression, and translation (Table S3). Next, we performed Gene Set Enrichment Analysis, and significantly enriched biological processes were clustered based on the cooccurrence of the same leading genes (Tables S4 and S5). Downregulated single biological processes obtained from proteomics and transcriptomics in USP21-KO cells allowed cluster formation. Functional enrichment analysis revealed that the most downregulated set of transcripts and proteomics are involved in mitochondrial ATP synthesis/oxidative phosphorylation (normalized enrichment score (NES) = − 3,22 and leading genes = 278 for transcripts; NES = −2,1 and leading proteins=58 for proteins) (Fig. 3, *A* and *B*). Detailed analysis of proteomic data allowed to visualize the dysregulated proteins engaged in mitochondrial function in USP21 KO cells [according to Human MitoCarta3.0 classification (35)] (Fig. 3*C*; Table S6) and assign specific function to them (Fig. 3*D*). On the contrary, transcriptomic data did not show a significant downregulation of transcripts involved in mitochondrial function, suggesting that USP21 regulates mitochondrial function on protein rather than transcript level (Fig. S2). Additionally, we found that 45% of all proteins with reduced expression in HAP-1 USP21 KO cells are represented by proteins belonging to mitochondrial ribosomes. This indicates that affecting mitochondrial function in USP21-KO cells may result from the disruption of the mitochondrial translation system.

FASTKD2 is a key posttranscriptional regulator of mitochondrial gene expression involved in mitochondrial RNA processing and assembly of the mitochondrial large ribosomal subunit (36). Our proteomic analysis revealed that FASTKD2 is one of the most downregulated protein in HAP-1 USP21 KO cells (Log2 change (WT *versus* USP21 KO) = −1,454091708) (Fig. 1*B*) while its expression increased upon STAT3 activation (Fig. S3). These results suggest that both USP21 and STAT3 activation are critical for FASTKD2 expression and FASTKD2-mediated translation of mitochondrial proteins.

Taken together, our results demonstrate that USP21 regulates the expression of mitochondrial proteins involved in mitochondrial ribosome biogenesis, functioning of respiratory chain complex, and ATP synthesis, therefore playing an essential role in cellular metabolism.

### USP21 is a critical factor for energy metabolism and mitochondrial respiration

**Figure 4:**
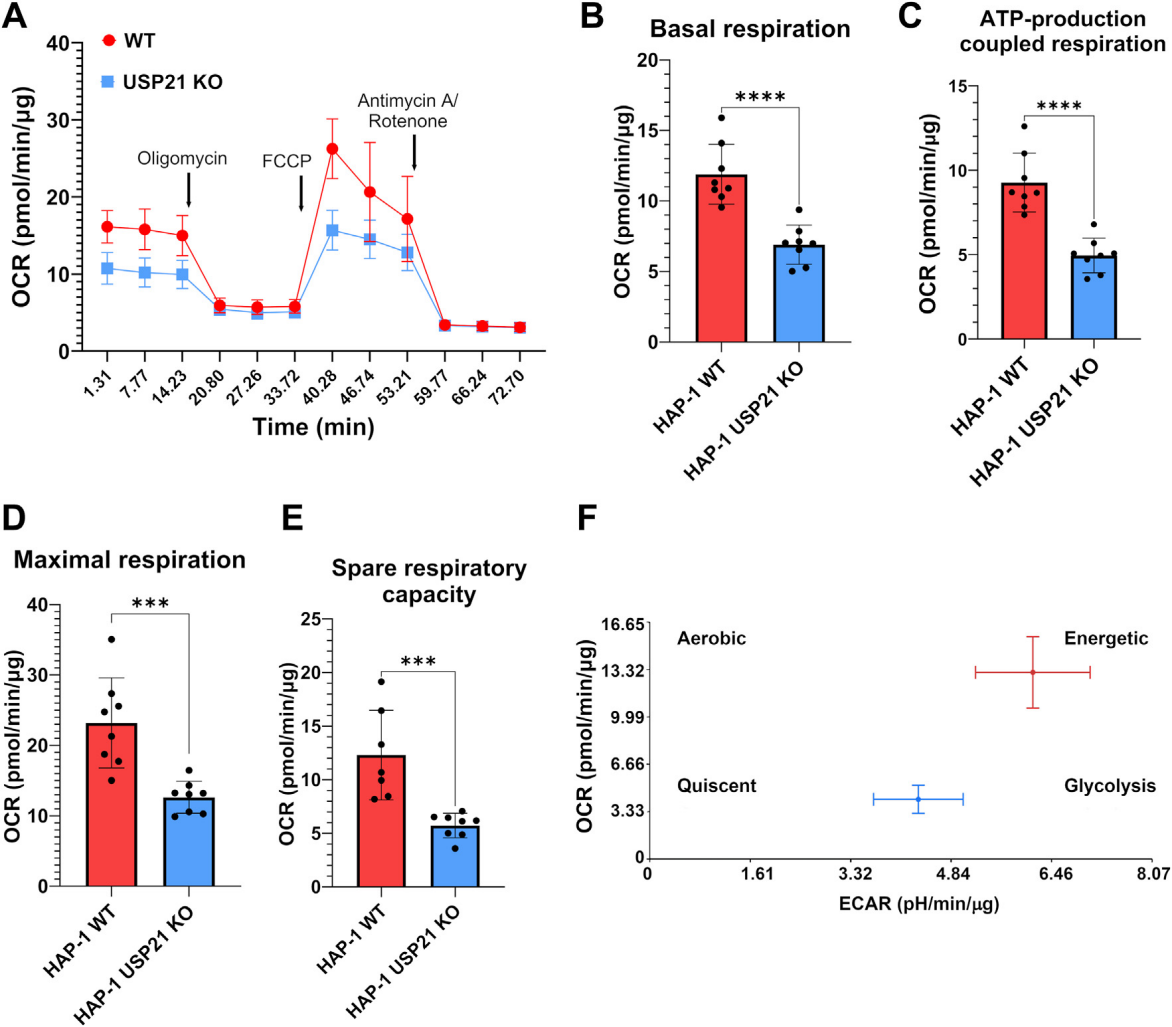
**USP21 is critical for mitochondrial function of cancer cells.** Cells were seeded at density 1 × 10^4^/well on Agilent Seahorse 96-well XF Cell culture microplate. Measurement of the kinetics of OCR in HAP-1 WT (*red*) and HAP-1 USP21 KO (*blue*) (*A*) was measured in real time after 72 h of cell culture. Parameters of mitochondrial function: ATP-production coupled respiration (*B*), basal respiration (*C*), maximal respiration (*D*), and spare respiratory capacity (*E*) were normalized to the total protein concentration. All seahorse data shown are compiled from two independent experiments using four technical replicates per experiment per condition and are presented as mean + std. errs. ∗∗∗*p* ≤ 0.001, ∗∗∗∗*p* ≤ 0.0001). Energy map of HAP-1 WT (*red*) and HAP-1 USP21 KO (*blue*) cells from representative experiment was obtained by charting mitochondrial ATP (OCR) *versus* glycolysis-generated ATP (ECAR) (*F*). ECAR, extracellular acidification rate; OCR, oxygen consumption rate; USP, ubiquitin-specific protease.

To investigate the effect of USP21 deletion on the bioenergetics of HAP-1 cells, we directly measured the oxidative potential in HAP-1 WT and USP21 KO cells using the seahorse extracellular flux analyzer (Fig. 4*A*). Detailed analysis of oxygen consumption rate (OCR) showed a 2-fold decrease of OCR for all mitochondrial respiratory parameters including, basal respiration, ATP production-linked respiration, maximal, and reserve respiratory capacity in USP21 KO cells in comparison to WT (Fig. 4, *B*–*E*). Final analysis of the OCR and extracellular acidification rate value ratio allowed us to characterize the difference in energy status of WT and USP21 KO cells as energy map. We demonstrated that the presence of USP21 maintained a high metabolic activity of HAP-1 WT cells, while the absence of USP21 shifted oxidative into anaerobic metabolism (Fig. 4*F*).

**Figure 5:**
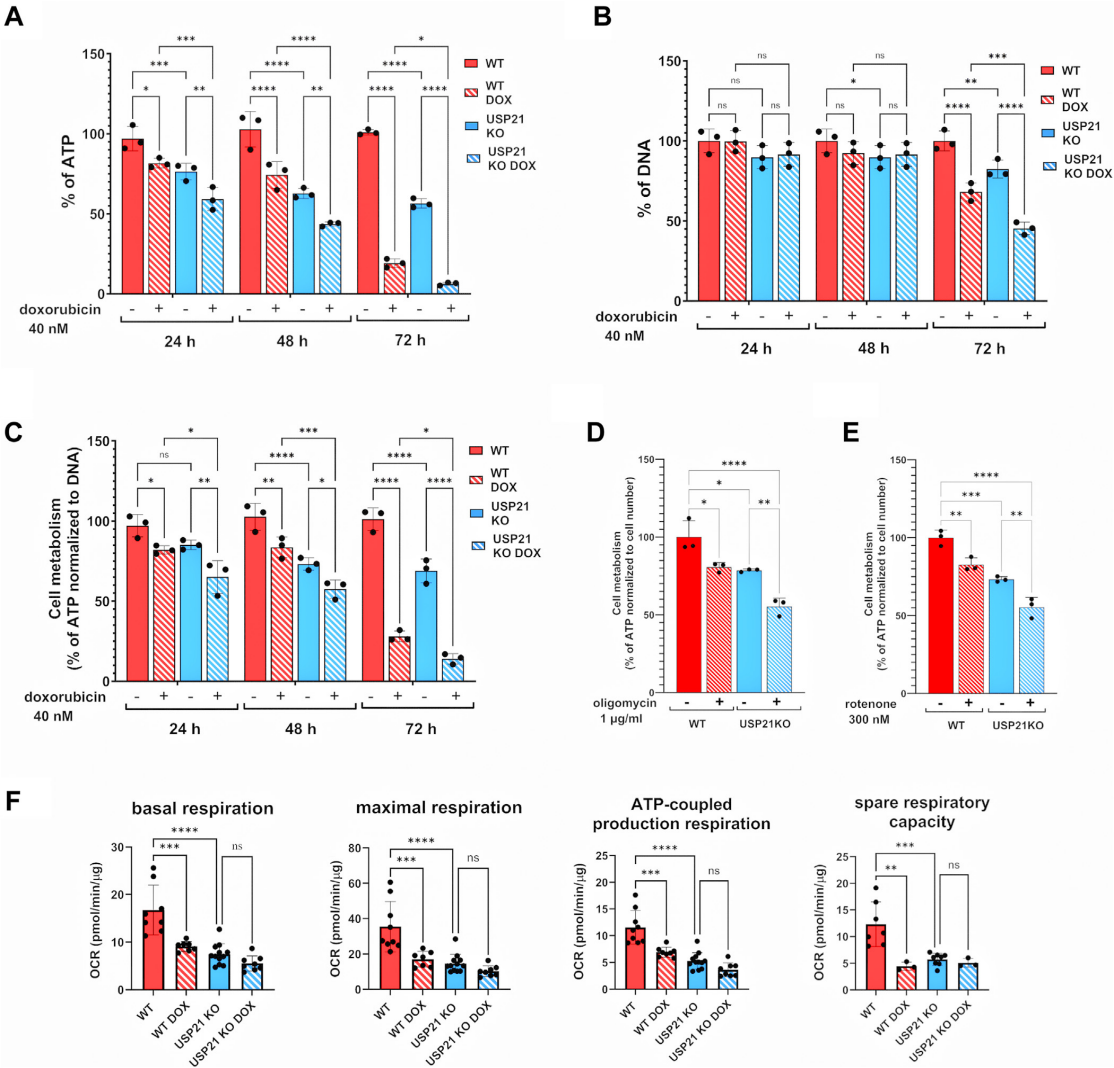
**Endogenous USP21 drives ATP production in healthy and damaged mitochondria of HAP-1 cell**. HAP-1 WT and HAP-1 USP21 KO cells were seeded at density 1 × 10^4^ on 96-well plate and cultured in the presence or absence of 40 nM doxorubicin. After 24, 48, or 72 h cells have been subjected to ATP level (*A*) and total cellular DNA content (*B*). Cellular metabolism was expressed as a percentage of cellular ATP level normalized to the total DNA level and referred to DOX-untreated WT cells (*C*). HAP-1 cells were cultured in the presence or absence of 100 μg/ml of oligomycin for 2 h (*D*) or 300 nM rotenone for 24h (*E*). Cellular metabolism was expressed as a percentage of cellular ATP level normalized by the initial cell number. Effect of treatment of HAP-1 WT and USP21 KO cells with 40 nM doxorubicin on mitochondrial respiratory function (basal respiration, maximal respiration, ATP-coupled production respiration, and spare respiratory capacity) was determined by measurement of OCR in seahorse XFe96 analyzer. Results were normalized to total cellular protein (*D*). Data were analyzed using ordinary one-way ANOVA. All data are presented as the mean ± SD (n = 3–8). *p* values were determined by Brown-Forsythe test. ∗∗*p* < 0.01; ∗∗∗*p* < 0.001; ∗∗∗∗*p* < 0.0001. DOX, doxorubicin; OCR, oxygen consumption rate; USP, ubiquitin-specific protease.

The mitochondria produce more than 90% of the total cellular ATP. The significantly reduced mitochondrial respiration in HAP-1 USP21 KO cells suggests that genetic deletion of USP21 affects mitochondrial bioenergetics. To verify this, we estimated the ATP level using an ATP-based luminescence assay that reflects the metabolic activity of the cells. Analysis of the difference in luminescence signal after 24 h of cell culture, reflecting the level of ATP, showed ca. 20% reduction in intracellular ATP content in USP21 KO compared to WT cells, which increased to approximately 40% and 50% after 48 and 72 h, respectively (Fig. 5*A*). In parallel, fluorescence measurement of total DNA was used to quantify cell number. While we did not observe any changes in DNA between the two genotypes, at the 24 h and 48 h time points of the HAP-1 culture, theUSP21 KO cells showed a significant decrease in DNA levels after 72 h. (Fig. 5*B*). These results demonstrate that deletion of USP21 affects cellular bioenergetics rather than direct proliferation of HAP-1 cells.

Next, we investigated the effect of USP21 deletion on mitochondrial bioenergetics and proliferation in the presence of doxorubicin (DOX). This approach was an attempt to assess the role of USP21 in sensitization of cancer cells to chemotherapeutic agents. DOX is a well-known chemotherapeutic agent that reduces cell proliferation by inhibition of DNA replication. DOX promotes the apoptosis resulting from mitochondrial dysfunction caused by oxidative stress and disruption of mitochondrial oxidative phosphorylation (37, 38, 39). We observed significant changes in the metabolic activity of WT and USP21 KO HAP-1 cells 24 h after treatment with 40 nM DOX, resulting in a decrease in ATP level (Fig. 5*A*), but no effect on proliferation compared to HAP-1 WT DOX-untreated control cells. Prolonged treatment of the cells with DOX (72 h) further highlighted changes between the two genotypes, with proliferation of the USP21 KO cells being completely inhibited at this time point (Fig. 5, *A* and *B*).

Then, we determined the role of USP21 in metabolic activity of HAP-1 cells treated with selective mitochondrial respiration inhibitors, oligomycin and rotenone. We found that oligomycin at 1 μg/ml reduced metabolic activity of HAP-1 WT cells to the level observed in USP21-depleted HAP-1 cells (ca. 75% metabolic activity), whereas metabolic activity of HAP-1 USP21 KO cells treated with oligomycin was reduced by 50% (Fig. 5*D*). Furthermore, we demonstrated that USP21 increased resistance of cancer cells to low dose of oligomycin (Fig. S3). Similar results were obtained for HAP-1 WT and USP21 KO cells treated with 300 nM rotenone, a mitochondrial electron transport complex I inhibitor (Fig. 5*E*). Taken together, normalized results indicate that lack of USP21 increases sensitivity of cancer cells to both nonspecific anticancer drug such as DOX and specific mitochondrial inhibitors, resulting in distortion of cellular bioenergetics and cell viability (Fig. 5, C–E).

Next, we focused on the comparison of mitochondrial respiration of WT and USP21 KO cells both in the presence and absence of DOX (Fig. 5*D*). Analysis of OCR changes indicated showed that the treatment of HAP-1 WT cells with 40 nM DOX for 24 h resulted in decrease in mitochondrial function, and the respiration rate decreased to a similar level in USP21 KO cells (Fig. 5*D*). Surprisingly, mitochondrial respiration parameters in USP21 KO cells treated with 40 nM DOX were not changed in comparison to untreated cells. This suggests that the DOX-induced reduction of ATP level is dependent on the USP21, which may be one of the mechanism by which DOX exerts its cytostatic effect (Fig. 5*D*; full and striped, blue bars).

## USP21 regulates the proliferation and migration of HAP-1 cells

**Figure 6:**
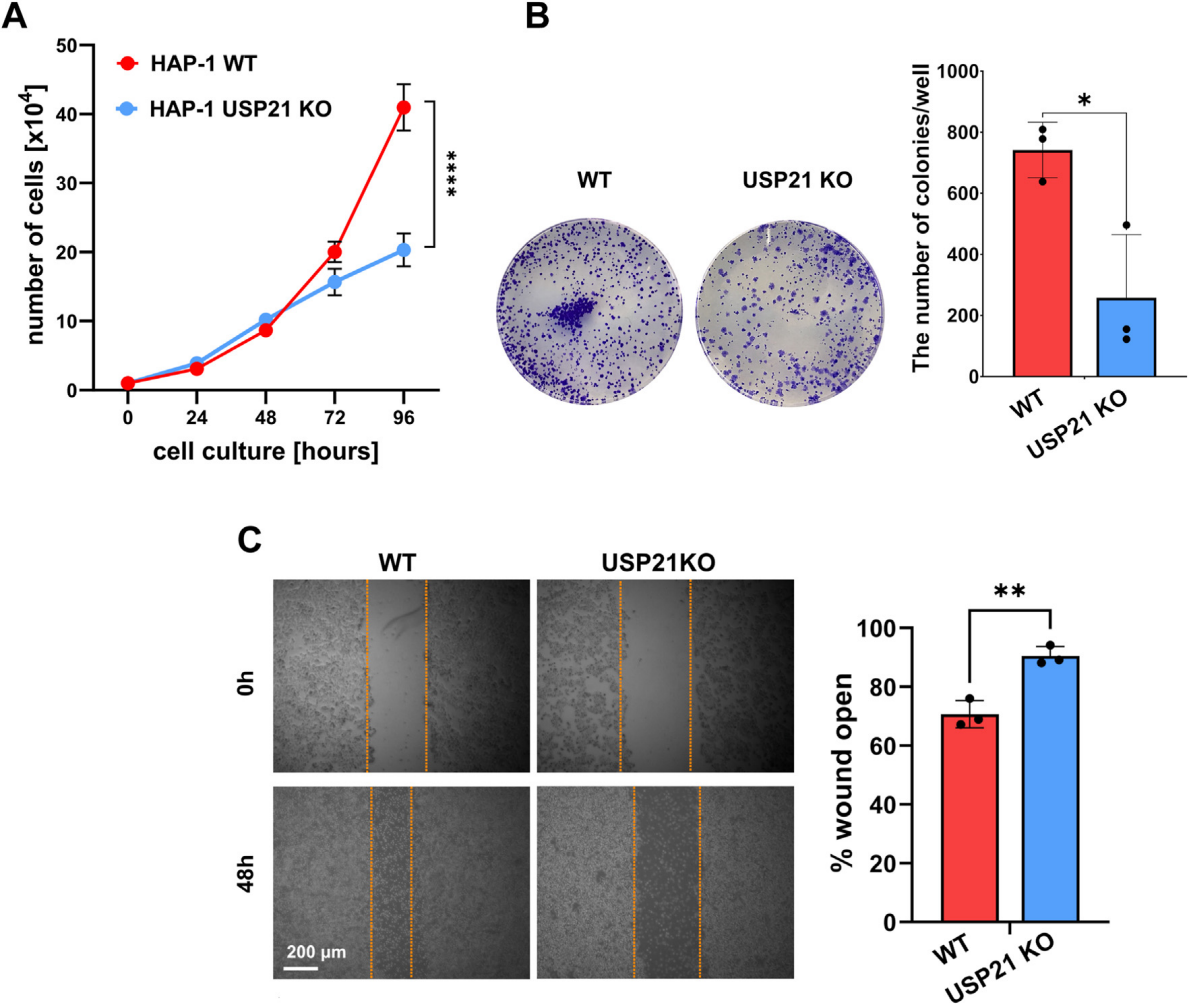
**USP21 drives HAP-1 proliferation and migration of HAP-1 cells.** Cell proliferation of HAP-1 WT (*red line*), HAP-1 USP21 KO (*blue line*) was analyzed by cell counting every day for 4 days. The number of live cells was determined automatically using Luna cell counter (*A*). The ability of cells in culture to grow was tested in the colony formation assay (*B*). HAP-1 WT and HAP-1 USP21 KO cells were placed 2 × 10^3^ cells in 12-well plates, incubated for 10 days, and the cells were stained with 0.1% crystal violet and counted (*left* panel). The mean number of colonies from 3 wells for HAP-1 WT (*red*) and HAP-1 USP21 KO (*blue*) are presented on the histogram (*right* panel). A wound healing assay was used to assess the migration ability of HAP-1 cells (*C*). The wound open area at 0 h and 48 h of cell culture was visualized (*left* panel, the scale bar represents 200 μm), measured using ImageJ software, and presented on the graph (*right panel;* HAP-1 WT*-red,* HAP-1 USP21 KO*- blue*). ∗∗*p* < 0.01, ∗∗∗*p* < 0.001, ∗∗∗∗*p* < 0.0001. USP, ubiquitin-specific protease.

Disturbance of mitochondrial function, ATP synthesis, and STAT3 phosphorylation observed in HAP-1 cells with genetically inactivated USP21 suggested that the fundamental features of cancer cells, such as proliferation and adhesion are regulated by USP21. To directly determine the role of USP21 on cell proliferation, HAP-1 WT and HAP-1 USP21 KO cells were cultured for 4 days at indicated time points (Fig. 6*A*). Analysis of the growth rate revealed that deletion of USP21 inhibited HAP-1 cell proliferation when cell density increased (Fig. 6*A*). A difference between two genotypes increased in time reaching a maximal value of 50% after 96 h (Fig. 6*A*). This is in line with our previous observation (Fig. 5) showing that USP21 deletion does not inhibit DNA synthesis but rather causes mitochondrial dysfunction in stressed environmental conditions. The USP21-dependent difference in cell proliferation was further examined in a colony formation assay. Counting the colonies on day 10 of seeding showed that colony formation by USP21-deficient cells was significantly (3-fold) reduced in comparison to WT cells (Fig. 6*B*). This result confirmed that USP21 plays an important role in the maintenance of proliferative capacity.

Migration is another process that depends on ATP and an additional functional assay was used to evaluate the role of USP21 in cancer development. Specifically, a wound healing assay was performed, revealing that USP21 KO cells had significantly inhibited cell migration compared to WT cells (Fig. 6*C*).

**Figure 7:**
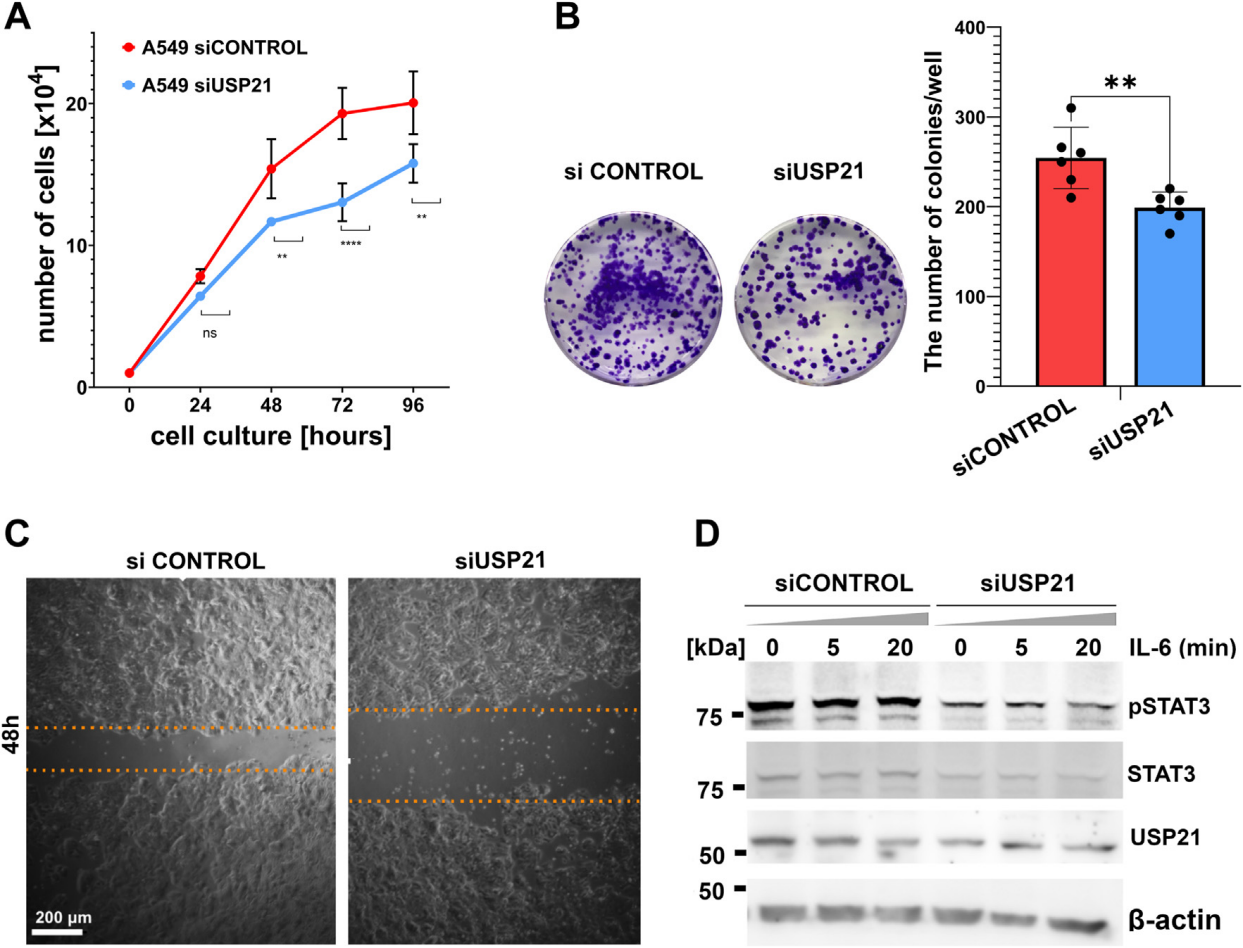
**USP21 drives proliferation, migration, and influences STAT3 phosphorylation in A549 cells**. Cell proliferation of A549 transfected with scramble siRNA-siCONTROL (*red line*) and siUSP21 (*blue line*), was analyzed by cell counting every day for 4 days. The number of cells was determined automatically using Luna cell counter (*A*). The ability of cells in culture to grow was tested in the colony formation assay (*B*). A549 cells treated with scramble siRNA and siUSP21 were placed 2 × 10^2^ cells in 24-well plates, incubated for 10 days, and the cells were stained with 0.1% crystal violet and counted (*left panel*). The mean number of colonies from six wells for scramble siRNA (*red*) and siUSP21 (*blue*) treated cells are presented on the histogram (*right panel*). A wound healing assay was used to assess the migration ability of A549 cells (the scale bar represents 200 μm) (*C*). Western blot analysis of STAT3 phosphorylation in IL-6-stimulated USP21 silencing A549 cells (*D*). ∗*p* < 0.05, ∗∗*p* < 0.01, ∗∗∗*p* < 0.001, ∗∗∗∗*p* < 0.0001. IL-6, interleukin 6; STAT3, signal transducer and activator of transcription 3; USP, ubiquitin-specific protease.

Presented results on HAP-1 cells indicate that USP21 regulates fundamental processes integral to tumor development and progression. To expand our claims of the role of USP21 in cancer biology we decided to analyze the effect of USP21 silencing on proliferation, migration, and colony formation in the A549 cell line of non–small cell lung cancer. All our results demonstrate that knockdown of USP21 in A549 cells reflects the phenotype of HAP-1 USP21 KO cells (Fig. 7). In addition, we found out that A549 cells showed high constitutive Tyr705 phosphorylation of STAT3 that was unchanged even upon IL-6 stimulation, while genetically reduced amount of USP21 decreased STAT3 phosphorylation both in unstimulated and IL-6 stimulated cells. This result indicates that USP21 is a critical factor for STAT3-mediated cellular processes involved in cancer progression, and this mechanism may be common for several types of cancer cells.

## Discussion

USP21 is known to deubiquitinate and stabilize several transcription factors such as YY1, FOXM1, EZH2, Nanog, and GATA3, promoting cancer cell proliferation, migration, and invasion and *in vivo* tumor growth (9, 13, 15, 17, 40, 41). STAT3 is one of the most commonly activated transcription factors involved in the expression of various target genes promoting tumorigenesis, and therefore is traditionally considered as a promising target for cancer therapy. STAT3 has been identified as a transcription factor positively regulating the expression of USP21 with two potential STAT3-binding sites within the USP21 promoter (42). Currently, two types of STAT3 inhibitors have been developed: direct binders to STAT3 domains and indirect, which inhibit the STAT3 signaling upstream by targeting JAKs, Src, and Abl (43). The inhibitors of JAKs are successfully used in the treatment of cancer and immune-mediated diseases (32), suggesting that indirect inhibition of STAT3 is a promising approach. In contrast, despite many efforts, effective therapeutic interventions directly targeting STAT3 to achieve potent antitumor effects in the clinic have not been identified and still need to be elucidated (31). Using a specific inhibitor to target STAT3 could prove to be a valuable strategy in cancer treatment, potentially yielding widespread clinical benefits. However, due to numerous difficulties, including serious toxic effects, poor therapeutic effects, and intrinsic and acquired drug resistance, development of STAT3 inhibitors remains challenging (44). Therefore, inhibiting the expression of STAT3-dependent genes by targeting its regulators may constitute a potential pathway in the therapy of many pathological conditions. In the present study, we demonstrate that genetic inactivation of USP21 gene regulates STAT3 activation. Additionally, we show that lack of USP21 regulates mitochondrial respiration determining metabolism of cancer cells to proliferate and migrate.

Our observations are in line with previous studies that linked USP21 with the STAT3 phosphorylation level, suggesting the role of USP21 in the regulation of activation of STAT3/FOXO1 pathway and glycolysis in esophageal cancer cells (45) and STAT3/FOXD1 in a process of self-renewal of mesenchymal glioblastoma stem cells (46). Recent studies revealed that USP21 affects JAK2/STAT3 axis in triple-negative breast cancer by indirect regulation of JAK2 stability (47). Our immunoprecipitation experiment indicates that USP21 directly interacts with STAT3 and positively regulates STAT3 expression level in cancer cells (Fig. 2). We provide further evidence for the relevance of STAT3-USP21 axis in the context of cancer metabolism. STAT3 may be involved in different USP21-mediated cancer progression pathways, such as USP21-YY1-SNHG16 axis in non–small cell lung cancer (9). Enhanced tyrosine phosphorylation of STAT3 in the presence of USP21 in HAP-1 and A549 cells suggests that USP21-dependent regulation of STAT3 signaling pathway is common for various cancer cells. Our results also demonstrate that direct interaction of USP21 with STAT3 stabilizes the level of STAT3 in cells, increases its activation, and in consequence, expression of STAT3-dependent proteins, including those involved in mitochondrial functions.

It is commonly known that cancer progression involves activation of cellular processes that depend on a change in mitochondrial metabolism initiated by the activation of STAT3 (24, 48, 49). Here, we show that USP21 influences STAT3-driven expression of proteins essential for mitochondrial function (Table S6). Thus, we provide evidence presenting USP21 as an attractive therapeutic target for cancer. Moreover, the recent discovery of the contribution of STAT3 activation in the transmission of intracellular signals of multiple cytokines promoting inflammation in fibrotic diseases (50), suggests that inhibition of USP21 can be potentially used as a therapeutic strategy to prevent the progression of pulmonary fibrosis. Furthermore, downregulation of USP21 may affect STAT3-dependent metabolic programming of inflammation-induced macrophages (51) and inflammasome activation (52, 53), allowing to assume a possible role of inhibition of USP21 and USP21-STAT3 pathway in anti-inflammatory therapy.

**Figure 8:**
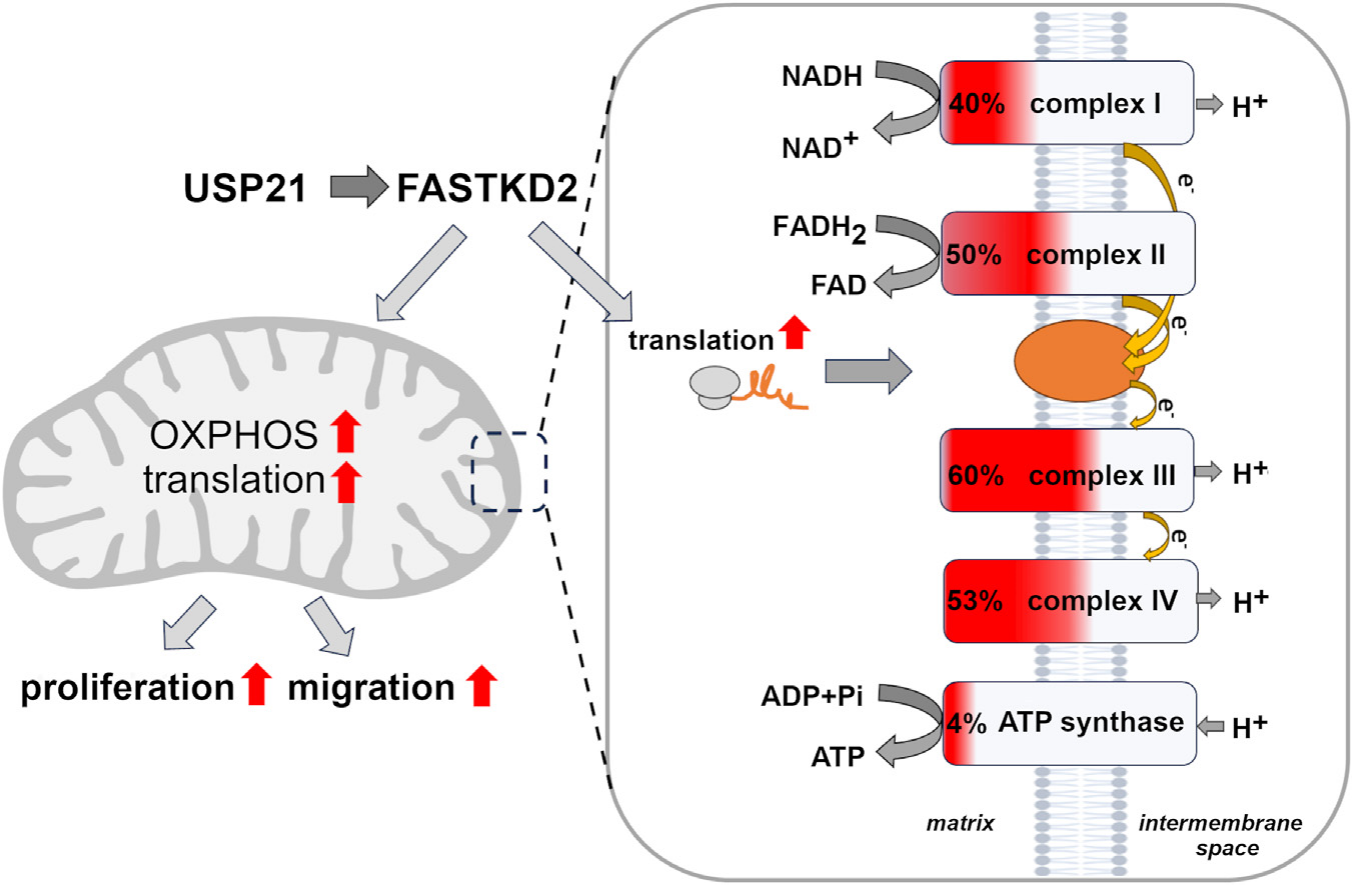
**Graphical visualization of the role of USP21 in oxidative phosphorylation and ATP production**. Graphical representation of downregulated proteins involved in the process of oxidative phosphorylation (OXPHOS) detected in HAP-1 USP21 KO compared to HAP-1 WT. USP21 stimulates expression of mitochondrial proteins as well as FASTKD2 which positively regulates translation of mitochondrial proteins belonging to the complexes of electron transport chain. The ratio of downregulated to the total components of the mitochondrial respiration chain complexes is expressed as a percentage and marked in *red*. Intracellular ATP produced during OXPHOS is used by cancer cells for proliferation and migration. USP, ubiquitin-specific protease.

Proteomic analysis revealed that deletion of USP21 caused a significant reduction in the level of mitochondrial proteins. We speculate that decreased level of proteins building mitochondrial ribosomes in USP21 deficient cells (Table S6, Fig. 8) may be directly linked to downregulated STAT3 activity, leading to disturbance of mitochondrial ribosome functioning and expression of mitochondrial genome-encoded proteins. Mitochondria require a specific transcription and translation machinery to synthesize a subset of mitochondrial proteins. This processes is driven by FASTKD2, a mitochondrial RNA-binding protein required for mitochondrial RNA processing, mitochondrial ribosome biogenesis, and translation (54). We observed down-expression of FASTKD2 in HAP-1 USP21 KO cells, which explains impaired biogenesis of mitochondrial ribosomes, translation of mitochondrial proteins, and compromised mitochondrial respiration in USP21-deleted cells (54, 55). We demonstrated that activation of STAT3 stimulates the expression of FASTKD2 (Fig. S4), suggesting the role of the USP21-STAT3 axis in the process of mitochondrial protein synthesis driven by FASTKD2. This mechanism needs to be further elucidated.

According to the canonical pathway, activation of STAT3 by tyrosine phosphorylation regulates nuclear gene expression involved in proliferation, survival, and self-renewal (24). However, the presence of a mitochondrial-pool of STAT3 which regulates mitochondrial function is also a widely described process (56, 57, 58, 59). The exact mechanism of STAT3 translocation into mitochondria has never been fully elucidated. Recently, it was demonstrated that acetylation of STAT3 promotes its translocation into mitochondria (60). STAT3 activation *via* phosphorylation at Ser727 is linked to enhancing the electron transport complexes in the mitochondria, resulting in increased ATP production (61, 62, 63). Our preliminary analysis of subcellular localization of STAT3 showed a decreased acetylation level of STAT3 in HAP-1 USP21 KO cells, which may explain the reduced level of STAT3 in the mitochondrial fraction (Fig. S5). Further explanation of the mechanism of USP21-dependent STAT3 translocation to mitochondria, leading to mitochondrial protein translation, can significantly expand our understanding of cancer metabolism.

Regulation of mitochondria function in an ubiquitin-dependent manner is relatively well described process in the context of stabilization of mitochondrial proteins (64, 65). Several DUBs such as USP8, USP14, USP15, USP30, USP33, and USP35 are located in the mitochondria and have an ability to regulate mitophagy and mitochondrial homeostasis, mainly by antagonizing Parkin activity (66, 67, 68, 69). In turn, located mainly in cytosol, USP2 was shown to regulate mitochondrial respiration and ATP production by maintaining the mitochondrial membrane potential and integrity (70). To date, knowledge about the role of USP21 in the mitochondrial function is limited only to skeletal muscle cells, where it has been shown that USP21 ablation causes increased oxygen consumption in mitochondria and ATP reduction (71). Similarly, we observed that deletion of USP21 in HAP-1 cancer cells decreased ATP level, but the effect on oxygen consumption was the opposite. The reduced energy state of HAP-1 USP21 KO cells is not the result of disruption of mitochondrial membrane potential or reduced mitochondrial mass (Figs. S6 and S7), but rather due to downregulation of proteins that are part of the respiratory chain complexes and involved in ATP synthesis (Tables S2 and S6). These results indicate that the control of cell metabolism by USP21 is a common process; however, the regulatory mechanisms are cell-specific.

Deficiency of mitochondrial proteins involved in cellular bioenergetics leads to changes in mitochondrial functioning, which may be used in the treatment of various diseases, including cancer (72, 73, 74). A growing amount of evidence suggests that mitochondrial bioenergetic metabolism may be important in controlling the function and proliferation of cancer cells. In contrary to the conventional point of view that mitochondrial metabolism is dispensable for tumor growth, recent works indicate that the mitochondria are active in most cancer cells and mitochondrial metabolism is essential for tumor growth especially in Kras-driven cancers (75, 76, 77, 78, 79, 80). Cancer progression depends on the energy from mitochondrial respiration, in particular ATP synthesized during the electron transport chain (81, 82, 83). Our findings present USP21 DUB as a positive controller of STAT3 activity and cancer metabolism. Furthermore, downregulation of USP21 may affect STAT3-dependent metabolic programming of inflammation-induced macrophages (51) and inflammasome activation (52, 53), allowing to assume a possible role of inhibition of USP21 and USP21-STAT3 pathway as a novel and attractive anticancer and anti-inflammatory therapeutic strategy.

## Experimental procedures

### Cell culture conditions

Both HAP-1 WT and USP21 KO (HAP-1 USP21 KO) cells were purchased from Horizon Discovery. HEK293 and A549 cell lines were purchased from the American Type Culture Collection. Human HAP-1 cells were cultured in Iscove-modified Dulbecco’s medium. A549 cells were cultured in the Roswell Park Memorial Institute Medium (RPMI 1640 medium). HEK293 cells were cultured in a Dulbecco's modified Eagle's medium (DMEM). All media were supplemented with 10% fetal bovine serum and 1% penicillin and streptomycin and were obtained from Invitrogen (Life Technologies). Cells were routinely passaged in 0.25% Trypsin-EDTA (HyClone) until reaching 85 to 90% confluence and were routinely tested for *Mycoplasma* contamination.

### Transfection of cells

Transfection of cells was used for silencing and overexpression of the USP21 gene. Transfection with siRNA was performed in 12-well or 96-well plates at cell confluence ∼70% using Lipofectamine 2000 (Invitrogen) in Iscove-modified Dulbecco’s medium or DMEM supplemented with 5% fetal bovine serum without antibiotics according to the manufacturer’s instruction. To obtain USP21 knockdown cells, silencing experiments were performed using USP21 small interfering RNA (USP21 Silencer select siRNA #4392420, Ambion) and nontargeting control siRNA (Negative Control Silencer select siRNA #4390843, Ambion). Cells were harvested 48 h after transfection and used for other experimental procedures. For the overexpression of USP21 in HAP-1 WT cells, transfection was performed using pcDNA3.1-His-HA-USP21 plasmid (GenScript) and Neon Transfection System (Thermo Fisher Scientific) according to the manufacturer’s instruction. Briefly, 100 μl of HAP-1 cells (1 × 10^6^), suspended in buffer R (Thermo Fisher Scientific), were mixed with 1 μg or 5 μg of pcDNA3.1-His-HA-USP21 plasmid and nucleofected using the following parameters: pulse voltage 1450 V, pulse width 10 ms, pulse number: 3. Subsequently, cells were suspended in 2 ml of medium and plated into 6-well plate. Cells were harvested 24 h upon nucleofection and used for further experimental procedures.

For immunoprecipitation assay, HEK293T cells were transiently transfected with pcDNA3.1-HA-USP21 (GenScript) and pCMV6-myc-STAT3 (OriGene) plasmids by polyethylenimine-based transfection method using 1:3 (DNA:polyethylenimine) ratio.

### STAT3 activation and immunoblotting

Cells (1 × 10^6^) were treated with IL-6 (50 ng/ml) at 37 °C for the indicated time periods followed by washing with PBS and harvesting. Cells were lysed in radioimmunoprecipitation assay buffer supplemented with protease/phosphatase inhibitors (Sigma-Aldrich) for 30 min at 4 °C and centrifuged at 15 000g 10’. Supernatants were collected and used to determine the total protein concentration (Pierce bicinchoninic acid protein assay, Thermo Fisher Scientific). Equal amounts of total protein were separated by 10% SDS-PAGE and transferred onto nitrocellulose membranes. Membranes were blocked in 3% bovine serum albumin for 2 h and then incubated overnight at 4 °C with following primary antibodies obtained from Cell Signaling Technology: mouse anti-STAT3, rabbit anti-phospho-STAT3 (Tyr705), mouse anti-β-actin, or from Invitrogen mouse anti-USP21. Next, the membranes were incubated with horseradish peroxidase-conjugated secondary antibodies for 2 h at room temperature. Proteins were visualized using enhanced chemiluminescence assay (Bio-Rad) and normalized to β-actin.

### Coimmunoprecipitation assay

HEK293T cells were cotransfected with pcDNA.3.1-HA-USP21 and pCMV6-Myc-STAT3 plasmids for 48 h and lysed in lysis buffer containing 50 mM Tris–HCl pH 7.5, 150 mM NaCl, 0.5% NP40, 1 mM DTT, 10 mM NEM (Sigma-Aldrich), protease, and phosphatase inhibitor cocktail (Sigma-Aldrich). Whole cell lysates were incubated overnight at 4 °C with beads conjugated to anti-HA (Thermo Fisher Scientific) antibody. Following three washes with the lysis buffer, the coprecipitated proteins were eluted by 2xLaemmli sample buffer, incubated for 5 min at 95 °C, and subsequently analyzed by immunoblotting using specific antibodies.

### Luciferase assay

pTF-STAT3-Luc reporter vector (BioCat) and pcDNA3.1-HA-USP21, or pcDNA3.1 plasmids were transfected into HEK293T cells by Lipofectamine 2000 (Invitrogen) according to the manufacturer’s instruction. Eight hours later, cells were treated with 50 ng/ml of IL-6 and incubated for 16 h. Then, cells were prepared for luciferase assay by using the Dual-Glo Luciferase Reporter Assay System (Promega) according to the manufacturer’s instruction.

### Cell growth and cellular metabolism measurement

Cells were seeded at 1 × 10^4^/well in a 96-well plate and cultured in the presence or absence of 40 nM DOX (Abcam), 1 μg/ml oligomycin (Sigma-Aldrich), and 300 nM rotenone (Sigma-Aldrich) within a certain time period. For measurement of cell growth, cells were trypsinized and stained with orange acridine (1:10 v:v ratio) at different time points. The number of cells was counted using Luna automated cell counter. Alternatively, cell proliferation was analyzed by measurement of total nucleic acids level using CyQUANT Cell Proliferation Assay Kit (Thermo Fisher Scientific), according to the manufacturer’s instruction. Cellular metabolism was analyzed using ATP-based CellTiter-Glo Luminescent assay (Promega) according to the manufacturer’s procedure. Absorbance and luciferase signal were measured using SpectraMax i3x Multi-Mode Microplate Reader. Cellular metabolism was expressed as a percentage of cellular ATP level normalized to the total DNA level and referred to DOX-untreated WT cells as a control.

### Wound healing assay

Wound healing assay was used to evaluate *in vitro* cell migration capacity. Cells were seeded at a density of 0.3 × 10^6^ into a 12-well plate and grown to confluence. For stimulating a wound, a sterilized 100 μl pipette tip was used to make a scratch. Images of wound healing were captured after 48 h using the Axiocam ERc 5s camera (Zeiss) and ZEN 3.5 Software (https://www.zeiss.com). Cell migration was quantified by measuring the area of the wound open with ImageJ software (https://imagej.net).

### Colony formation assay

A volume of 1 ml of complete medium containing 2 × 10^3^ HAP-1 and 2 × 10^2^ A549 cells was placed in each well of a 12-well plate and 24-well plate, respectively. After 10 days, cells were washed with PBS, fixed with 3% paraformaldehyde, and stained with 0.25% crystal violet for 20 min. The number of colonies was counted by ImageJ software.

### Proteomic sample preparation and LC-MS/MS analysis

For proteomic analysis of HAP-1 WT and USP21 KO cells were seeded on the 6-well plate at density 0.5 × 10^6^/well and 1 × 10^6^ USP21 KO/well, respectively, and cultured for 24 h. Cells were lysed in 2% SDS in 50 mM Hepes pH 8.0. The supernatant was collected, and the protein concentration was determined using a Bicinchoninic acid assay (Pierce). Subsequently, 50 μg of protein per sample was precipitated with chloroform/methanol, resuspended in 100 mM Hepes pH 8.0 containing 5 mM tris(2-carboxyethyl)phosphine and 10 mM chloroacetamide, and subjected to overnight digestion at 37 °C with sequencing grade modified trypsin (0.5 μg). Next, the samples were acidified with trifluoroacetic acid to the final concentration of 1% and centrifuged at 12 000*g* for 3 min at room temperature. Tryptic peptides (10 μg per sample) were then desalted with the use of AttractSPE Disks Bio C18 (Affinisep), tandem mass tag (TMT)-labeled on the solid support (84), compiled into a single TMT sample and concentrated using a SpeedVac concentrator. Peptides in the compiled sample were fractionated (8 fractions) using the basic reversed-phase fractionation. Prior to liquid chromatography with tandem mass spectrometry (LC-MS/MS) analysis, the peptide fractions were resuspended in 0.1% trifluoroacetic acid, 2% acetonitrile in water. Chromatographic separation was performed on an Easy-Spray Acclaim PepMap column 15 cm long × 75 μm inner diameter (Thermo Fisher Scientific) at 35 °C by applying acetonitrile gradients in 0.1% aqueous formic acid at a flow rate of 300 nl/min. Each basic reversed-phase peptide fraction was measured twice, using a shorter (90 min) and a longer (120 min) LC-MS/MS method. An UltiMate 3000 nano-LC system was coupled to a Q Exactive HF-X mass spectrometer *via* an easy-spray source (all Thermo Fisher Scientific). The Q Exactive HF-X was operated in TMT mode with survey scans acquired at a resolution of 60,000 at m/z 200. Up to 18 of the most abundant isotope patterns with charges 2 to 5 from the survey scan were selected with an isolation window of 0.7 m/z and fragmented by higher-energy collision dissociation with normalized collision energies of 32, while the dynamic exclusion was set to 35 s. The maximum ion injection times for the survey scan and the MS/MS scans (acquired with a resolution of 30,000 at m/z 200) were 50 and 96 ms, respectively. The ion target value for MS was set to 3e6 and for MS/MS to 1e5, and the minimum automatic gain control target was set to 1e3.

### Proteomic data processing and analysis

The data were processed with MaxQuant v. 1.6.17.0 (https://www.maxquant.org/) (85), and the peptides were identified from the MS/MS spectra searched against the UniProt reference human proteome (UP000005640) using the build-in Andromeda search engine. Cysteine carbamidomethylation was set as a fixed modification and methionine oxidation, glutamine/asparagine deamidation, and protein N-terminal acetylation were set as variable modifications. For *in silico* digests of the reference proteome, cleavages of arginine or lysine followed by any amino acid were allowed (trypsin/P), and up to two missed cleavages were allowed. Reporter ion MS2-based quantification was applied with reporter mass tolerance = 0.003 Da and min. reporter PIF = 0.75. The false discovery rate (FDR) was set to 0.01 for peptides, proteins, and sites. Match between the runs was enabled. Other parameters were used as preset in the software. Unique and razor peptides were used for quantification enabling protein grouping (razor peptides are the peptides uniquely assigned to protein groups and not to individual proteins). Reporter intensity corrected values were uploaded into Perseus v. 1.6.10.0 (https://maxquant.net/perseus/) (86). Standard filtering steps were applied to clean up the dataset: reverse (matched to decoy database), only identified by site, and potential contaminants (from a list of commonly occurring contaminants included in MaxQuant) protein groups were removed. The intensity values were log2 transformed and protein groups with all 6 values were kept. The values were then normalized by median subtraction within TMT channels. Student’s *t* test (two-sided, permutation-based FDR = 0.01, S0 = 0.1) was performed on the dataset to return protein groups, which levels were statistically significantly changed in USP21 KO vs WT samples. For lists of proteins identified and quantified in USP21 KO *versus* WT total cell extracts, see Table S1. This dataset has been deposited to the ProteomeXchange Consortium (87) *via* the PRIDE (88) partner repository with the dataset identifier PXD 044105.

### Transcriptomic data analysis

Transcriptomic analysis was performed by the Genomics Core Facility at the Centre of New Technologies (University of Warsaw) in accordance with the applicable procedures. Briefly, RNA samples were isolated from HAP-1 WT and HAP-1 USP21 KO cells using RNeasy Plus Kit (Qiagen) and sequenced using the Illumina NovaSeq 6000 system. Raw sequences were trimmed according to quality using Trimmomatic version 0.39 (http://www.usadellab.org/cms/?page=trimmomatic) (89) using default parameters, except MINLEN, which was set to 20. Trimmed sequences were mapped to the human reference genome provided by ENSEMBL, (version grch38_snp_tran) using Hisat2 with default parameters. Optical duplicates were removed using the MarkDuplicates tool from the GATK package version 4.1.2.0 (https://gatk.broadinstitute.org/) (90). Reads that failed to map to the reference were extracted using Samtools (http://samtools.github.io) (91) and mapped to the Silva meta-database of rRNA sequences (version 119) (92) with SortMeRNA version 2.1b (https://sortmerna.readthedocs.io/) (93) using “–best 1” option. Mapped reads were associated with transcripts from the grch38 database (Ensembl, version 106) using HTSeq-count (version 0.11.2) with default parameters. Differentially expressed genes were selected using the DESeq2 package (version 1.16.1) (94) and presented as a volcano plot. The dataset has been deposited to the Sequence Read Archive (SRA) with accession number PRJNA1026098 (https://www.ncbi.nlm.nih.gov/bioproject/PRJNA1026098).

### Functional enrichment

For both transcriptomic and proteomic data Gene Set Enrichment Analysis was performed for GO (The Gene Ontology Consortium 2019) gene sets and Kyoto Encyclopedia of Genes and Genomes gene sets using ClusterProfiler package (95), FDR was applied on enriched terms *p*-values due to multiple hypotheses testing. Pairwise semantic similarity analysis and clustering were done by the GOSemSim package version 2.24.0 (https://bioconductor.org/packages/GOSemSim/) (96) using Jiang and Conrath’s distance metric (97). Pairwise similarities of the enriched terms were clustered using a hierarchical clustering approach. The obtained clusters were then redefined based on the processes assigned to each cluster manually. For each cluster median of the NES coefficient was calculated to obtain information about the direction of regulation.

### Seahorse assay

HAP-1 WT and HAP-1 USP21 KO cells were seeded at density 1 x 10^4^/well and in Seahorse XF96 polystyrene tissue culture plates (Agilent Technologies) for 72 h. To examine the effect of DOX, cells were treated with 40 nM DOX (Abcam) 24 h prior to measurement. Immediately before the measurement, the cells were incubated in Seahorse XF DMEM assay medium (Agilent Technologies) in a non-CO_2_ incubator at 37 °C for 30 min. OCR was measured every 5 min with mixing using Seahorse XFe96 Analyzer (Agilent Technologies). Cell Mito Stress Test was used for assessing mitochondrial function. Finally, 1.5 μM oligomycin (Sigma-Aldrich), 0.75 μM carbonyl cyanide 4-(trifluoromethoxy)phenylhydrazone (FCCP) (Sigma-Aldrich), and the 0.5 μM mixture of rotenone/antimycin A (Sigma-Aldrich) were injected sequentially according to the instrument setting procedure, and then the data analysis of the OCR linked to ATP production, maximal respiration, and spare respiratory capacity was carried out (98). The data were normalized according to the total protein content in each well.

### Statistical analysis

In this study, we used GraphPad Prism version 9 (www.graphpad.com) to analyze the data. Each experiment was repeated at least three times independently. The experimental data were expressed as the mean ± SD. The data analysis used in the article included two-tailed Student’s t-tests or two-way ANOVAs.

## Data availability

All data supporting the findings of this study are available within the article and its Supplementary Information. Transcriptomics data were deposited into the Sequence Read Archive (SRA) under accession number PRJNA1026098 and are available at the following URL: https://www.ncbi.nlm.nih.gov/bioproject/PRJNA1026098. The mass spectrometry proteomics data have been deposited to the ProteomeXchange with identifier PXD 044105.

## Supporting information

This article contains supporting information.

## Conflict of interest

The authors declare that they have no conflicts of interest with the contents of this article.
