# Supplementary material for: The ubiquitin-specific protease 21 is critical for cancer cell mitochondrial function and regulates proliferation and migration
Source: J Biol Chem. 2024 Sep 19;300(10):107793. doi: 10.1016/j.jbc.2024.107793 (PMC11513602; doi:10.1016/j.jbc.2024.107793)
Supplement: Supporting Information [file mmc1.docx]

**Supporting information**

**Supplemental experimental procedures**

**Mitochondrial mass measurement**

HAP-1 WT and HAP-1 USP21 KO were seeded at 1x10^4^/well density and growth in 96-well black plates for 24 h before staining. Next, cells were stained with the use of 20 nM MitoTracker Green FM (Thermo Fisher Scientific) and Hoechst H33342 (5 µg/ml) for cell imaging. Cells were incubated in the dark, in optimal growth conditions for 45 min. Afterwards, cells were washed with PBS and analyzed using ImageXpress Pico cell imaging system (Molecular Devices) or flow cytometry (CytoFlex S, Beckman Coulter). For flow cytometry analysis cells were stained with the Zombie Aqua dye (BioLegend) for determining the living cells population. Only live cells (Zombie negative) were used for the measurement of Mean Fluorescence Intensity (MFI) and further analysis. For imaging analysis, the Integrated Density for green channel was calculated using ImageJ. For calculation, 5 random areas of the full photo were analyzed and 10 random ROIs were assigned in each area. The ROIs (Regions Of Interest) were assigned manually on the basis of nucleus with the margin.

**Mitochondrial membrane potential measurement**

The mitochondrial membrane potential was analyzed JC-9 dye (Thermo Fisher Scientific) fluorescence measurement using flow cytometry and fluorometer approaches. HAP-1 WT and HAP-1 USP21 KO cells were seeded in 96-well plates at density 1x10^4^/well. After 24 h of culture HAP-1 WT cells were transfected with USP21 siRNA or non-targeting siRNA. For flow cytometry analysis, after 48h from silencing the cells were trypsinized and incubated with 5 µM JC-9 dye in culture medium for 15 min at 37 °C. As a positive control, cells were incubated with 5 µM FCCP and 2 µg/ml valinomycin for 10 min at 37 °C, before JC-9 staining. Next, cells were washed three times with medium followed by three washes with PBS and suspended in 200 µl of PBS. Next, the cells were stained with the Zombie Aqua stain to determine the living cell population. Only live cells (Zombie negative) were further analyzed for Mean Fluorescence Intensity (MFI). For fluorescence microplate reader cells were incubated with JC-9 for 15 minutes under optimal growth conditions. Then, cells were washed three times with full medium, following three washes with PBS. For fluorescence measurement attached cells were supplemented with 200 µl of PBS. Red and green fluorescence was measured in excitation/emission 485 nm/530 nm and 535 nm/590 nm, respectively, using Spectramax i3x microplate reader (Molecular Devices). For the positive controls, cells were incubated with 5 µM FCCP and 2 µg/ml valinomycin for 10 min at 37 °C, before JC-9 staining.

**Subcellular fractionation**

HAP1-WT and HAP-1 USP21 KO cells (10x10^6^) after stimulation by IL-6 (50 ng/ml) for 16h were harvested and washed once with PBS. Pelleted cells were resuspended in 500 μl of fractionation buffer (FB: 20 mM Hepes, pH7.4, 10 mM KCl , 2 mM MgCl2, 1 mM EDTA, 1 mM EGTA) containing freshly added protease inhibitors and 1 mM DTT and incubated on ice for 15 min. The cell suspension was passed through a 27 G needle 10 times and left on ice for 20 minutes. Cellular lysates were centrifuged at 720xg for 5 min to pellet nuclei (N fraction). The supernatant containing mitochondria and cytoplasm was transferred into a fresh tube and centrifuged at 10000xg for 10 min. Supernatant containing cytoplasm (C fraction) was collected to a fresh tube, while pelleted mitochondria were washed two times in FB and resuspended in TBS/0.1% SDS (M fraction). All fractions were subjected to western blot analysis.

**Supplemental figures and legends**

**Fig. S1**

**
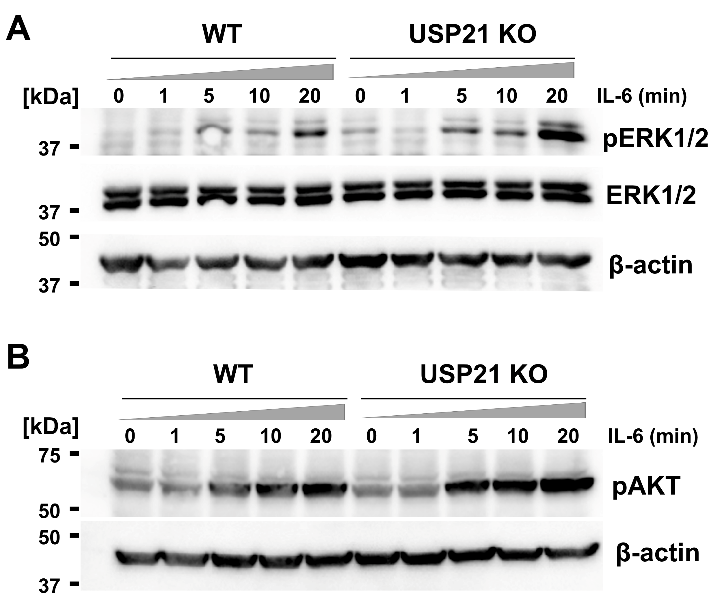
**

**Fig. S1. USP21 does not affect MAPK/ERK and AKT signaling pathways in HAP-1 cells.** Western blot analysis of ERK (A) and AKT(B) phosphorylation in IL-6 stimulated HAP-1 WT and HAP-1 USP21 KO cells.

**Fig. S2**

**
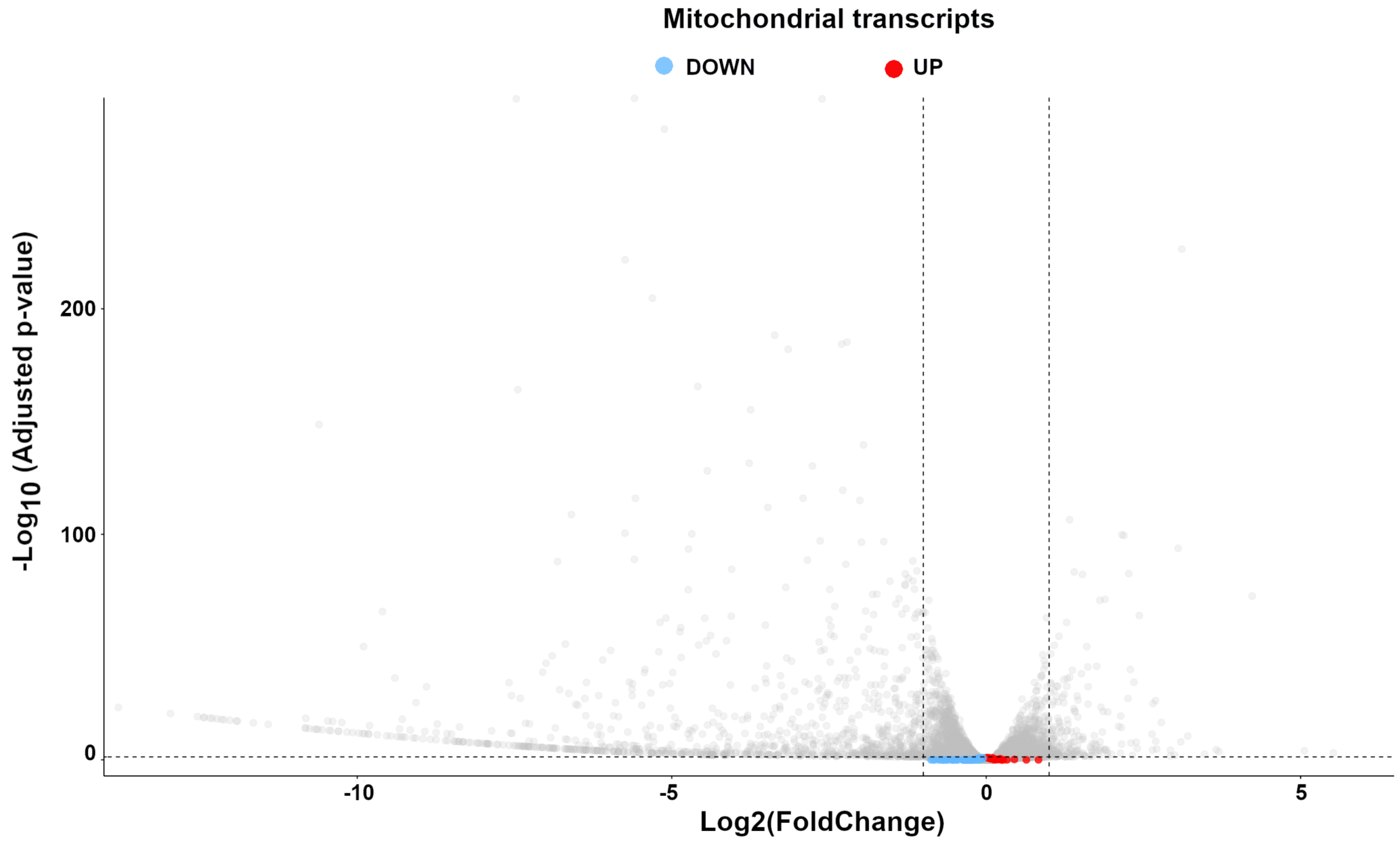
**

**Fig. S2. USP21 is not essential for expression of transcripts involved in mitochondrial function in HAP-1 cells.** Downregulated (*blue*) and upregulated (*red*) transcripts in HAP-1 USP21 KO cells involved in mitochondria functioning overlaid on volcano plot depicting no significant differences of transcript levels in total cell extracts from USP21 KO *vs* WT cells.

**Fig. S3.**


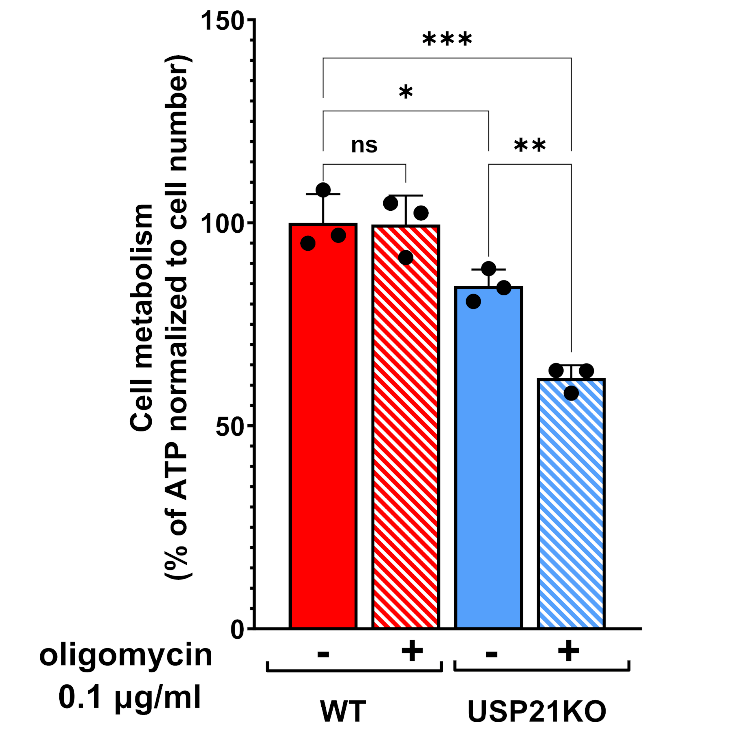


**Fig.S3. Endogenous USP21 increases resistance of HAP-1 cells to low concentration of mitochondrial respiration inhibitor.** HAP-1 WT (*red*) and HAP-1 USP21 KO (*blue*) cells were seeded at density 2x10^4^ on 96-well plate and cultured in the presence or absence of 0.1 μM oligomycin (*red, blue dashed bars*). After 2 h cells have been subjected to ATP level. Cellular metabolism was expressed as a percentage of cellular ATP level normalized to the number of cells and referred to oligomycin-untreated HAP-1 WT cells.

**Fig. S4**


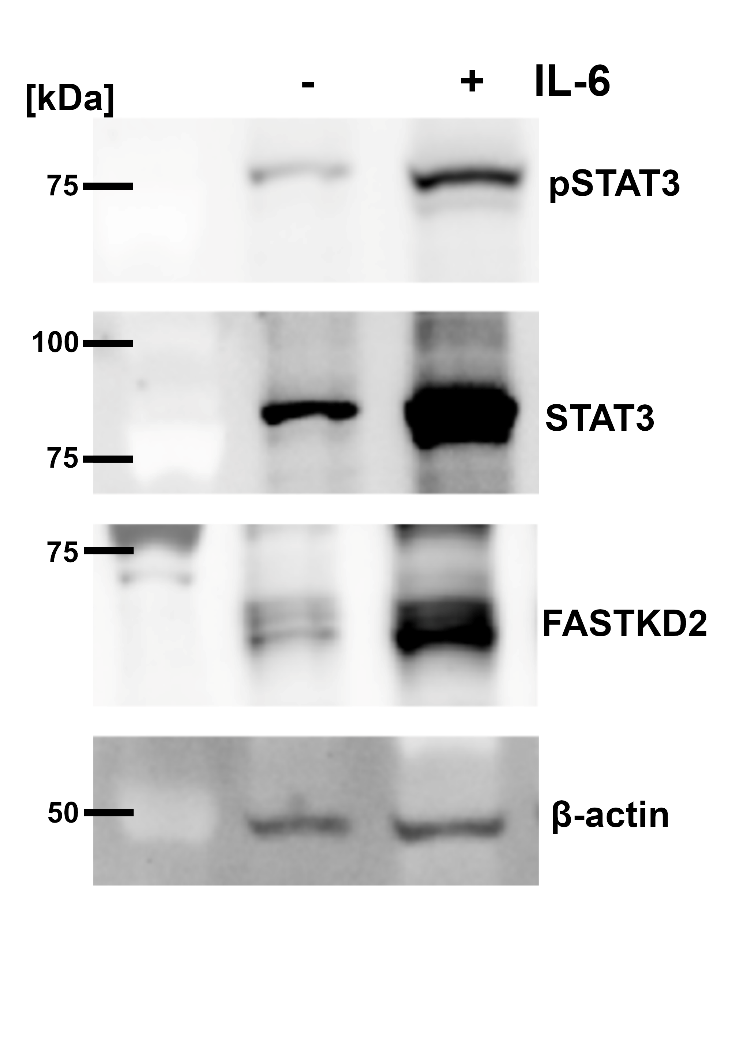


**Fig. S4. Expression of FASTKD2 depends on the STAT3 activation.** Western blot analysis of FASTKD2 and STAT3 expression level in unstimulated and IL-6 stimulated HAP-1 WT cells for 16h.

**Fig. S5.**


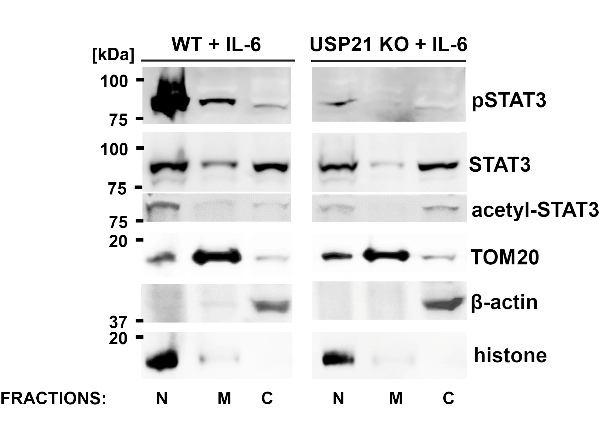


**Fig. S5. Western blot analysis of subcellular localization of STAT3 in HAP-1 cells.** Nuclear (N), mitochondrial (M) and cytoplasmic (C) fractions were prepared from HAP-1 WT and HAP-1 USP21 KO cells activated with IL-6 for 16h. STAT3 localization was determined by immunoblotting with antibodies recognized phosphotyrosine 705 on STAT3, STAT3 acetylated at lysine 685 (Cell Signaling), and total STAT3. Individual fractions were detected using following antibodies: histone H3 (Cell Signaling)- nuclear fraction; β-actin (Cell Signaling) – cytosolic fraction; TOM20 (Cell Signaling)- mitochondrial fraction.

**Fig. S6.**


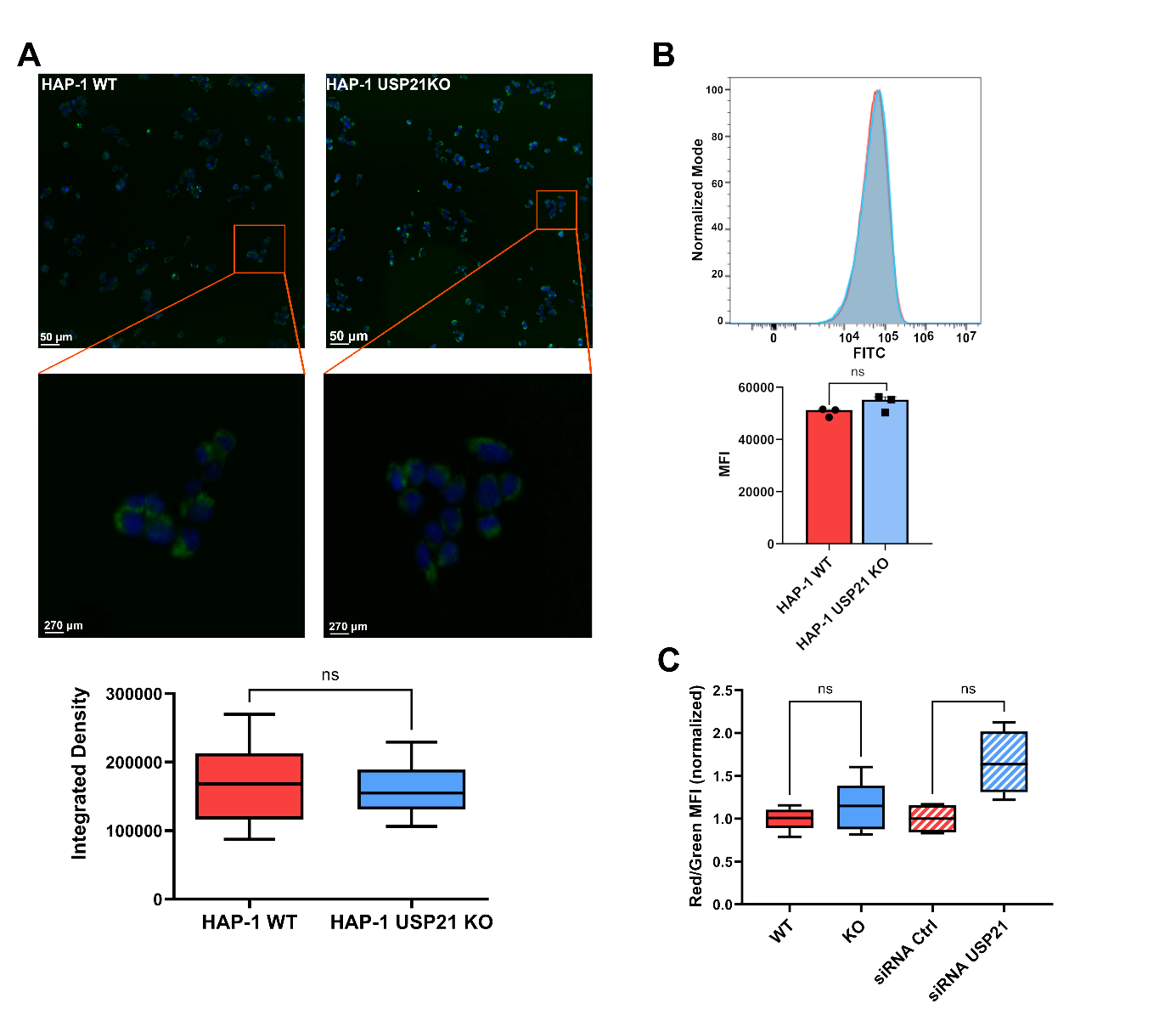


**Fig. S6. Deletion of USP21 does not change mitochondrial mass and mitochondrial membrane potential.** The effect of USP21 depletion on mitochondrial mass was determined by mitochondria staining with MitoTracker Green (20 nM) and visualized in ImageXpress Pico cell imaging system (*upper panel*) (scale bar is 50 μm). Orange square was enlarged by a scale factor 5,4. The integrated density of green channel is presented in a graph as a mean of MitoTracker Green intensity (*lower panel*) (A). Flow cytometry analysis of mitochondrial mass in HAP-1 WT (*red*) and HAP-1 USP21 KO (*blue*) cells are presented as an overlay histogram (*top panel*) and quantification of mean MitoTracker Green FM intensity (*bottom panel*) for (B). Data presented as mean ± SEM (n = 5), statistically not significant. Flow cytometry analysis of mitochondrial membrane potential changes in HAP-1 cells: WT, USP21 knock-out (USP21 KO), silenced with control siRNA (siCONTROL) and siRNA targeting USP21 (siUSP21) using JC-9 staining (C). The values of JC-9 green/red fluorescence ratio determined by flow cytometry for HAP-1 WT (*red*), HAP-1 USP21 KO (*blue*) and HAP-1 treated with control siRNA (*red, striped*), and siUSP21 (*blue, striped*) are expressed as a mean fluorescence intensity. Data are referenced as average ±SD of four experiments.

**Fig. S7.**


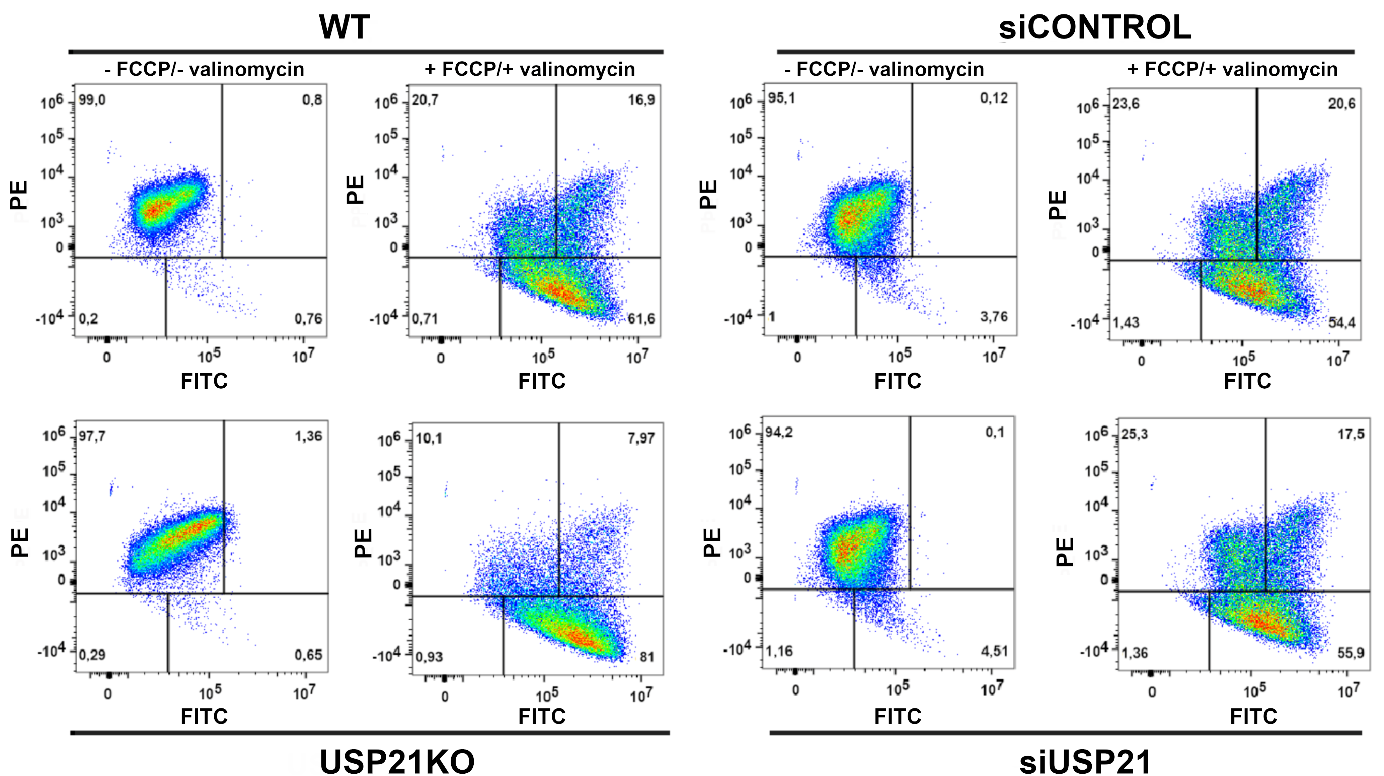


**Fig. S7. USP21 does not change mitochondrial membrane potential**. Representative scatter plots showing flow cytometry analysis of mitochondrial membrane potential changes in HAP-1 WT, HAP-1 USP21 KO, and USP21 knockdown HAP-1 (siUSP21) cells using JC-9. Red (PE) and green (FITC) fluorescence of mitochondria stained with JC-9 in control and FCCP/valinomycin treated cells. Note that cells with depolarized mitochondrial membrane (treated with FCCP/valinomycin) shift from the center of dot plot to the bottom right quadrant.

**Supplementary Tables:**

**Supplementary table 1.** Transcriptomic analysis of dysregulated proteins in HAP-1 USP21 KO *vs.* HAP-1 WT cells

**Supplementary table 2.** Proteomic analysis of dysregulated genes in HAP-1 USP21 KO *vs*. HAP-1 WT cells

**Supplementary table 3.** Functional Enrichment and clustering analysis of transcriptome (a) and proteome (b) of HAP-1 USP21 KO vs HAP-1 WT cells

**Supplementary table 4.** Functional and cluster analysis of transcriptome of HAP-1 USP21 KO vs HAP-1 WT cells

**Supplementary table 5.** Functional and cluster analysis of proteome of HAP-1 USP21 KO vs HAP-1 WT cells.

**Supplementary table 6.** The list of downregulated and upregulated proteins involved in mitochondrial function, including mitochondrial ribosome formation (*red*) and electron transport chain (green) in HAP-1 USP21 KO cells. The downregulated proteins whose expression is dependent on STAT3 (according to the ENCODE Transcription Factor Targets Dataset) are highlighted in yellow.
