# Supplementary material for: The ubiquitin-specific protease 21 is critical for cancer cell mitochondrial function and regulates proliferation and migration
Source: J Biol Chem. 2024 Sep 19;300(10):107793. doi: 10.1016/j.jbc.2024.107793 (PMC11513602; doi:10.1016/j.jbc.2024.107793)
Supplement: Supplementary Table S3 [file mmc4.docx]

| **GO ID** | **Transcripts downregulated in USP21 KO** | **NES** |
| --- | --- | --- |
| GO:0098742 | Cell-cell adhesion via plasma-membrane adhesion molecules | -4.31 |
| GO:0045333 | Cellular respiration | -4.10 |
| GO:0009060 | Aerobic respiration | -4.08 |
| GO:0140053 | Mitochondrial gene expression | -4.03 |
| GO:0007156 | Homophilic cell adhesion via plasma membrane adhesion molecules | -3.95 |
| GO:0006091 | Generation of precursor metabolites and energy | -3.94 |
| GO:0015980 | Energy derivation by oxidation of organic compounds | -3.84 |
| GO:0032543 | Mitochondrial translation | -3.77 |
| GO:0006119 | Oxidative phosphorylation | -3.68 |

| **GO ID** | **Proteins downregulated in USP21 KO** | **NES** |
| --- | --- | --- |
| GO:0032543 | Mitochondrial translation | -2.96 |
| GO:0140053 | Mitochondrial gene expression | -2.91 |
| GO:0019646 | Aerobic electron transport chain | -2.34 |
| GO:0042773 | ATP synthesis coupled electron transport | -2.32 |
| GO:0042775 | Mitochondial ATP synthesis coupled electron transport | -2.32 |
| GO:0006119 | Oxidative phosphorylation | -2.2 |
| GO:0022904 | Respiratory electron transport chain | -2.19 |
| GO:0042776 | proton motive force-driven mitochondrial ATP synthesis | -2.11 |
| GO:0009060 | Aerobic respiration | -2.11 |

Supplementary table 3.
